# Sol-Gel Behavior of Hydroxypropyl Methylcellulose (HPMC) in Ionic Media Including Drug Release

**DOI:** 10.3390/ma4101861

**Published:** 2011-10-24

**Authors:** Sunil C. Joshi

**Affiliations:** School of Mechanical and Aerospace Engineering, Nanyang Technological University, Singapore 639 798, Singapore; E-Mail: mscjoshi@ntu.edu.sg; Tel.: +65-6790-5954; Fax: +65-6791-1859

**Keywords:** HPMC, hydrogel, surfactants, hofmeister series, thermogelation

## Abstract

Sol-gel transformations in HPMC (hydroxypropyl methylcellulose) are being increasingly studied because of their role in bio-related applications. The thermo-reversible behavior of HPMC is particularly affected by its properties and concentration in solvent media, nature of additives, and the thermal environment it is exposed to. This article contains investigations on the effects of salt additives in Hofmeister series on the HPMC gelation. Various findings regarding gelation with salt ions as well as with the ionic and non-ionic surfactants are presented. The gel formation in physiological salt fluids such as simulated gastric and intestine fluids is also examined with the interest in oral drug delivery systems. The processes of swelling, dissolution and dispersion of HPMC tablets in simulated bio-fluids are explored and the release of a drug from the tablet affected by such processes is studied. Explanations are provided based on the chemical structure and the molecular binding/association of HPMC in a media. The test results at the body or near-body temperature conditions helped in understanding the progress of the gelation process within the human body environment. The detailed interpretation of various molecule level interactions unfolded the sol-gel mechanisms and the influence of a few other factors. The obtained test data and the established mathematical models are expected to serve as a guide in customizing applications of HPMC hydrogels.

## 1. Introduction

Researchers have shown particular interest in the behavior of hydroxypropyl methylcellulose (HPMC), chemically presented as C_6_H_7_O_2_(OH)_x_(OCH3)_y_(OC_3_H_7_)_z_ with x + y + z = 3, where aqueous solutions of these carbohydrate polymers have revealed gel reversibility with temperature [1]. Due to its high swellability and thermal gelation properties, HPMC has, until now, been the most important carrier material for the drug release systems [2,3,4]. Cellulose and its derivates in the form of agglomerated porous particles are regarded as the most useful filler for direct-compression tablets [5,6]. They are physically stable under normal conditions. They are chemically inert to the active ingredients, compatible with packing components and easily available [7]. It is expected that the studies on HPMC gelation in a biocompatible media would provide a better understanding of HPMC’s role in drug delivery.

Many techniques are available for studying the sol-gel transitions in HPMC hydrogels. These processes primarily include dynamic light scattering [8], differential scanning calorimetry (DSC) [9,10], rheological measurements [11,12] and nuclear magnetic resonance (NMR) [13,14].

This article presents studies on the gelation processes for HPMC in various ionic media. The ability of typical salting-out and salting-in salts in affecting the thermogelation of HPMC is systematically studied. The enthalpy and entropy changes (ΔH and ΔS respectively) determined from the DSC curves are discussed along with the contributions from the salt ions in the Hofmeister series. The findings for the HPMC solutions with the salt as well as with ionic and non-ionic surfactant additives are presented. Gel formation in physiological salt fluids such as simulated gastric and intestinal fluids (SGF and SIF respectively) is examined to have a better insight into the oral drug delivery systems.

## 2. Materials, Sample Preparation and Equipments Used

Three different grades of HPMC, as listed in Table 1, were procured from Sigma-Aldrich, Inc., USA.

**Table 1:** Specifications of hydroxypropyl methylcellulose (HPMC) powders used.

Sample	Molecular weight M_n_	Methyl (CH_3_) substitution (%)	Hydroxypropyl (CH_2_CHOHCH_3_) substitution (%)	Viscosity (2 wt% aqueous solutions at 25 °C)
A	10,000	60–67%	7–10%	6 cp
B	22,000	28–30%	7–12%	40–60 cp
C	86,000	60–67%	7–12%	4,000 cp

Various salts in Hofmeister series were procured for studying their salting-in and salting-out effects on the gel formation. Analytical grade salts, purchased from Sino Chemical Co. Ltd., Singapore, included monovalent salts (NaCl, KCl, NaBr, and NaI), divalent salts (Na_2_HPO_4_, K_2_HPO_4_, and Na_2_SO_4_) and a trivalent salt (Na_3_PO_4_), which were used as received. In addition, sodium hydroxide (NaOH) and monobasic potassium phosphate (KH_2_PO_4_) required for preparing the buffer solutions and the hydrochloric (HCl) acid (37%) were purchased from Fluka Chemical Corp., WI, USA. Surfactants, sodium n-dodecyl sulfate (SDS), sodium n-decyl sulfate (SDeS) and Triton X-100 were purchased from Sigma-Aldrich Inc., USA. Sodium n-hexadecyl sulfate (SHS) was ordered from Alfa Aesar-A Johnson Matthey Company, MA, USA. Indomethacin, the drug that was loaded into HPMC tablets where needed, was procured from Aldrich, USA. To prepare the aqueous solutions, de-ionized (DI) water from a Millipore (MA, USA) Alpha-Q water-purifying system was used as obtained at room temperature and at different thermal conditions.

All materials were stored in a controlled humid environment. The powders were dried overnight at 60 °C and stored in a desiccator before use. The readily prepared aqueous solutions were stored immediately in a refrigerator (at 4 °C) for 24 hours so as to obtain homogeneous and transparent mixtures before their use for testing.

Various calorimetric measurements were conducted using a micro-differential scanning calorimeter (VP-DSC, MC-2 microcalorimeter, MicroCal Inc., USA). A 0.5158 mL of sample solution and an equal amount of reference fluid (deionized water or other solution as required) were hermetically sealed into the sample cell and the reference cell, respectively. DSC curves for cooling and heating (at a rate of 1.0 °C /min) were recorded in the temperature range from 20 to 90 °C.

A control-strain rheometer (ARES 100FRTN1, Rheometric Scientific Inc., NJ, USA) was used to measure the flow properties and dynamic viscoelasticity of gel solutions. The rheometer was equipped with two sensitive force transducers for torque ranging from 0.004 to 100 g cm. The sample solution was poured onto the parallel-plate geometry (50 mm in diameter) and a small amount of silicone oil was applied at the periphery of the solution to prevent evaporation. The dynamic storage modulus (G′) and loss modulus (G″) were examined as the functions of temperature.

Micro-structural changes during and after the gel formation were examined using a confocal microscope (Axiotron 2, Carl Zeiss MicroImaging Co. Ltd., Germany) coupled with a hot plate (CSS 450, Linkam Scientific Instruments Ltd., Surrey, UK). A UV-vis spectrometer (Jasco V-570, Nippon-Bunko Co. Ltd., Tokyo, Japan) was used for light transmittance measurement through the sol-gel media at various temperatures, where the heating rate was controlled by a Peltier-type temperature controller.

The number average molecular weights (Mn) of the polymers were determined by gel permeation chromatography (GPC) (Waters 2690, Waters Corp., MA, USA) with a differential refractometer detector (Waters 410, Waters Corp., MA, USA). The mobile phase used was DI water with the flow rate of 1 mL/min. The weight and number average molecular weights were calculated from a calibration curve using a series of Dextran standards (Aldrich, USA, with molecular weight ranging from 667 to 778,000).

The HPMC tablets for studying their behaviour in bio-environment were prepared using the direct-compression method without any binder in Carver Laboratory Press (2158 series, Carver Inc., IN, USA).

## 3. HPMC in Aqueous Media with Hofmeister Series Salts

### 3.1. Hofmeister Series

Salts, including the common salt as discussed earlier, are known to influence the temperature-induced phase transitions in aqueous solutions of thermosensitive polymers, such as MC (methyl cellulose), poly(ethylene oxide)-poly(propylene oxide)-poly(ethylene oxide) triblock copolymers, triblock copolymers of poly(ethylene glycol) (PEG) and poly(D,L-lactide-co-glycolide) (PLGA), and copolymers of *N*-isopropylacrylamide [15,16,17,18]. Kim *et al*. [15] investigated the effects of salt on the thermogelation behavior of PEG-PLGA-PEG. They found that a salting-out salt, NaCl, decreased the gelation temperature through its water-structure formation properties. On the other hand, salting-in salts, such as NaSCN, increased the gelation temperature through their water-structure breaking properties. It is well known that the influence of ions follow the Hofmeister series, which originates from the abilities of ions to precipitate proteins [19]. The effect of anions decreases in the order SCN^−^ < ClO_4_^−^ < I^–^ < NO_3_^−^ < Br^−^ < Cl^–^ < F^–^ < H_2_PO_4_^−^ < S_2_O_3_^2–^ < SO_4_^2−^, whereas the influence of cations increases in the order Li^+^ > Na^+^ > K^+^ > Mg^2+^ > Ca^2+^ > Ba^2+^. However, the effect of cations is less significant than that of anions [20]. The anions on the right in the series are destabilizing ones or kosmotropes, whereas those on the left are the stabilizing ions or chaotropes. Various mechanisms and theories have been proposed to explain the effects of the Hofmeister series [20,21,22,23,24,25]; most of them are based on the capability of an ion to make or break the water structure.

Collins [21] suggested that an ion’s effect on water structure was due to the competition between water–ion interactions, whereas Omta *et al*. [24] reported that the presence of ions does not change the structure of a bulk water. This difference in explanation was possibly due to the ionic concentration and the sensitivity of the methods to the bulk structural changes. An alternative explanation is that the ions disrupt the hydration process at the water–solute interface [26]. New theories, such as specific ion binding, have also been proposed to explain the Hofmeister series [17,18].

### 3.2. Investigations Using DSC

Although the effects of salt on some sol-gel systems, including MC, have been previously investigated [16,27,28], few have reported about the effects of salt on the gelation of HPMC, even though HPMC has more applications in pharmaceutics and bio-fields.

**Figure 1:**
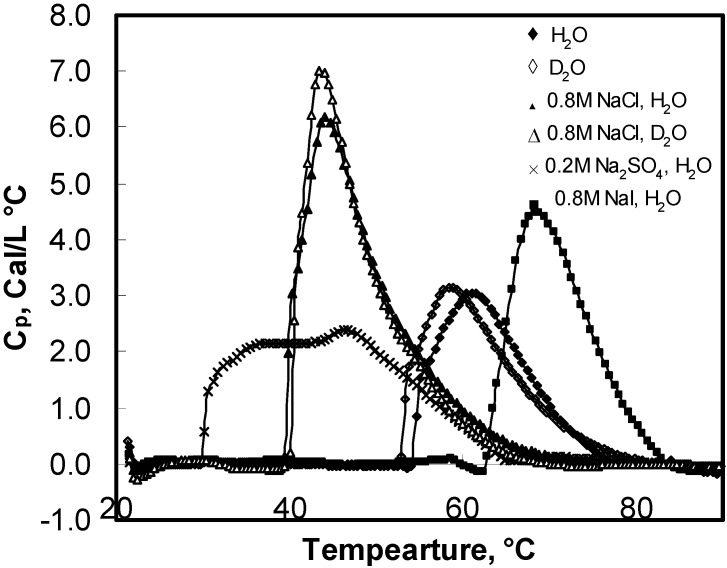
Differential scanning calorimetry (DSC) scans showing Cp plots for HPMC solutions in H_2_O and D_2_O with various salts.

**Figure 2:**
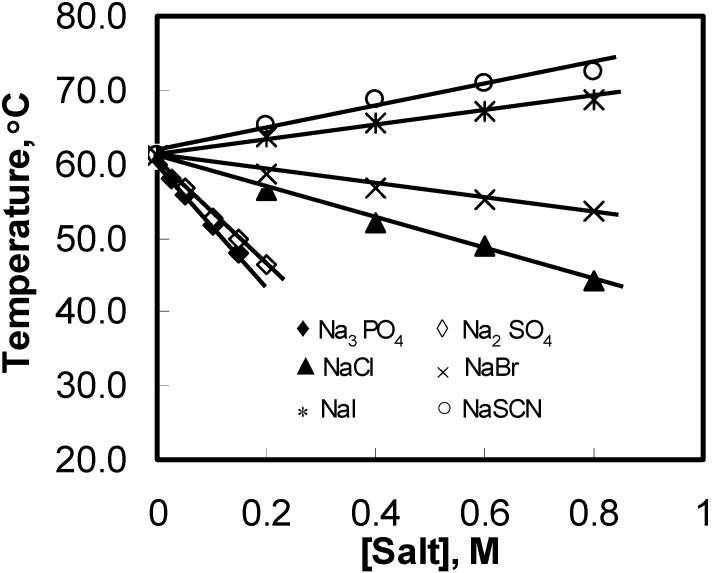
Peak temperature (T) variations for aqueous solutions of 1 wt% HPMC as a function of the added salts concentration during the heating process.

Mitchell *et al*. [29] showed that various salts that lower the cloud point of HPMC gels follow the order of the Hofmeister series. Since turbidity measurement method has many limitations; microcalorimetry, which is a more powerful technique, is used to study the salting mechanism for HPMC gels. As seen in Figure 1, addition of NaCl shifted the DSC plot leftward whereas the presence of NaI in the HPMC solution caused the DSC curve to shift towards the right. Thus, NaCl led to the decrease in gelation temperature indicating salting-out phenomenon [15] whereas NaI was the cause for the salting-in phenomenon that led to the increase in the gelation temperature. The plots in Figure 2 show a linear decrease in the peak temperature ( T ) with the concentration of salt in the solutions. This indicates that all ionic effects have a linear dependence on the salt concentration.

The magnitude of T lowered with the addition of kosmotropic anions and increased in the presence of chaotropes. Moreover, the ability of ions to change the values of T depended strongly on the anions and was characterized with the slope ( κ ) of the fitted straight line (see Figure 2). The more negative the κ value was, the stronger the salting-out effect observed. PO_4_^3−^ had the strongest salting-out effect amongst all of investigated anions, and T decreased sharply with a negative κ of −86.0 °C/M. The slopes for the other three kosmotropic anions (SO_4_^2–^, Cl^–^, and Br^–^) were −72.6, −20.6, and −9.2 °C/M, respectively. The negative slope for the kosmotropic anions indicated that they promoted the sol–gel transition of the HPMC solutions. In contrast to kosmotropic anions, the slopes of chaotropic anions such as I− and SCN− were positive, and they were 9.4 and 14.3 °C/M, respectively, which indicated that they retarded the thermal gelation of the HPMC solutions. The results showed that the effects of anions on T of the HPMC solutions followed the sequence of the Hofmeister series.

As most studies have pointed out and demonstrated that the ions exert their salting-out/salting-in effects via their water structuring capability and not via the direct interactions between the ions and the polymer chains [30,31,32]; a similar mechanism is used to explain the Hofmeister series in this study.

The effects of ions on water structure are attributed to the competitions between ion-water interactions and water-water interactions [21]. The former interactions are dominated by charge density, and the latter interactions are dominated by hydrogen bonding. The ability of halide to lower $T_{m}$ decreases with its size (Table 2, regression coefficient = 1.0) [33].

**Table 2:** Linear correlation between κ of the fitted curves and the entropy of hydration (ΔS_hydr_), viscosity B coefficient of the anions, the anion radius. Note: ΔS_hydr_, viscosity B coefficient, and anion radius are from reference [30].

Ions	κ (°C/mol)	ΔS_hydr_ (J/K mol)	Viscosity B coefficient (L/mol)	Radius (Å)
PO_4_^3−^	−86.0	−421	0.495	2.38
SO_4_^2−^	−72.6	−200	0.206	2.30
Cl^−^	−20.6	−75	−0.005	1.81
Br^−^	−9.2	−59	−0.033	1.96
I^−^	9.4	−36	−0.073	2.20
SCN^−^	14.3	–	−0.103	2.13

Because the chloride ion has the smallest radius and largest charge density among the halides investigated in this study, it has the strongest ability to compete for water molecules and form hydrogen bonds with water molecules, which minimizes or weakens water-polymer hydrogen bonding. On the other hand, the low charge density of ions such as I^–^ provides the weakest competition for water molecules, which thus leads to salting-in effects and increased the solubility of the polymer.

Hribar *et al*. [22] argued that the effect of salt on the degree of water structuring is determined mainly by the entropies of ion salvation and viscosity changes. Anions with more negative entropy values are considered more effective in arranging water molecules in an orderly manner. A plot of the κ values versus the corresponding ΔS_hydr_ of the various salts anions yielded a straight line with a regression coefficient of 0.84, indicating that these two parameters were not well correlated linearly. On the other hand, viscosity B coefficient was used to quantify the degree of water structuring because it was related to the viscosity of an aqueous salt solution. Anions with a large viscosity B coefficient value exhibited strong water structure making capabilities. The regression coefficient for the κ verses viscosity B coefficient of the anions plots was found to be 0.91.

Even though the linear correlation between slopes κ (for T ) and ΔS_hydr_ and viscosity B coefficient was not well-defined, most anions followed a sequence. These seem to demonstrate that different mechanisms were involved in thermogelation of HPMC in presence of salts [34].

An alternative explanation proposed by Hribar *et al*. [22] is that water structure is determined by the balance between electrostatics and hydrogen bonding. Multivalent anions and the smallest anions such as F^−^ and Cl^−^ cause a strong electrostatic orientation of water molecules with respect to the anion, which makes water structure more ordered.

The figure below illustrated the physical structures of HPMC hydrogels in respectively 0.2 M Na_2_SO_4_ and 0.2 M NaI solutions. The figure legends are as follows: HPMC main chain (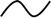); water molecules (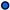); hydrophobic substitution (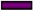); hydroxyl group (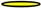); ions (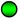); hydrophobic interaction (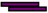); interchain hydrogen bonding (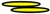).

**Figure 3:**
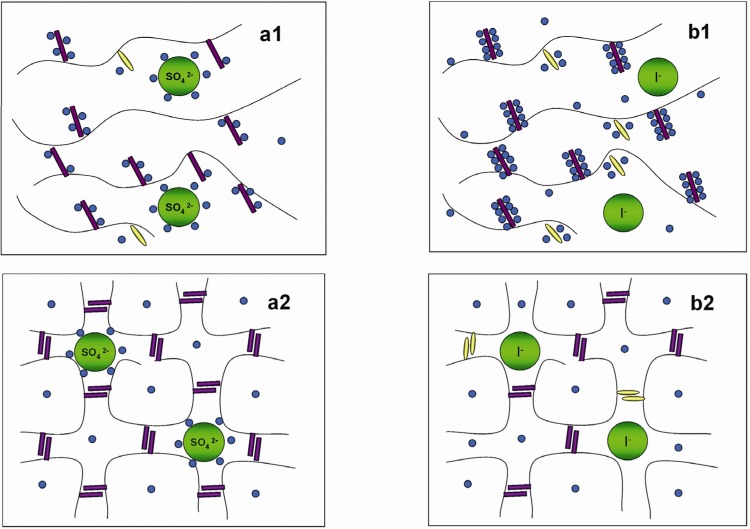
Physical structures of HPMC hydrogels in (**a**) 0.2 M Na_2_SO_4_ in H_2_O; (**b**) 0.2 M NaI in H_2_O (a1, b1 at lower temperatures and a2, b2 at higher temperatures).

As a result of more ordered water structure, a less number of water molecules are freely available to solvate the polymer chains. This facilitates and accelerates the hydrophobic association of methyl substitutions causing reduction in T of the HPMC solutions (refer to Figure 3(a1,a2)). In contrast, large monovalent anions such as I^−^ and SCN^−^ have low charge densities, and water structures around them are less organized. Having no restraint on their movement, these water molecules tend to increase hydrogen bonding between them and the polymer, which results in an increase in T of the HPMC solutions as shown schematically in Figure 3(b1,b2).

On the basis of these analyses, it is clear that both mechanisms could be applied to interpret the results in this study. The trivalent anion (PO_4_^3−^) was more effective in the salting-out effects than the divalent anion (SO_4_^2−^), followed by the monovalent anions such as Cl^−^. However, the trends did not relate well to the radius of the ions (refer Table 2). This is a strong indication that the valency was a dominating factor in comparison to the anionic radius.

**Figure 4:**
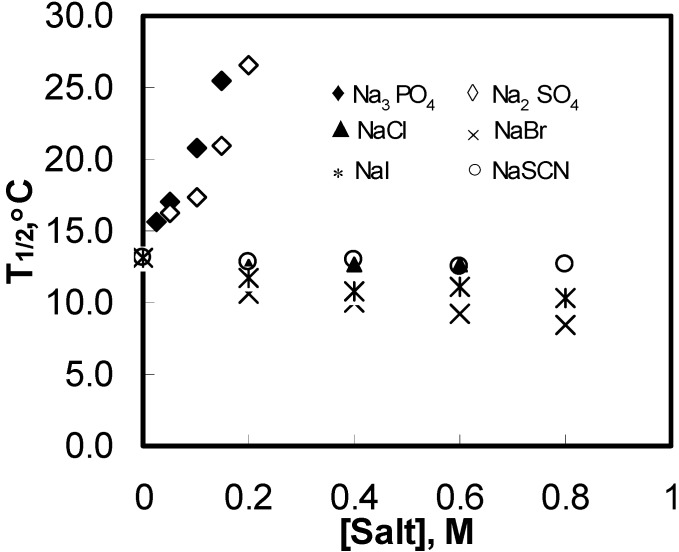
$T_{1/2}$ variations for aqueous solutions of 1 wt% HPMC as a function of the added salts concentration during the heating process.

Multivalent anions such as PO_4_^3−^ and SO_4_^2−^ had a strong salting-out effect as compared to monovalent anions. It was interesting that the pattern of thermograms of HPMC in the presence of SO_4_^2−^ was much broader than that of monovalent anions (Figure 1). The corresponding $T_{1/2}$ is illustrated in Figure 4.

$T_{1/2}$ for all monovalent anions was almost constant. It rose sharply with increasing concentration of a salt with multivalent anions. The results suggest that the effect of monovalent anions was more cooperative, whereas that of multivalent anions was less cooperative. Multivalent anions have strong ability to compete for water molecules in a solution. It is, however, unlikely that all such ions could have competed for water molecules at the water-HPMC interfaces because of their large size and tetrahedral coordination. Additionally, the ring structure of HPMC would have made it difficult for the multivalent anions to approach the water cages. Therefore, it may be construed that the water cages were weakened to a different extent by multivalent anions than they were by the monovalent anions. In other words, the strength of the water cages had a larger polydispersity in the presence of multivalent anions.

**Figure 5:**
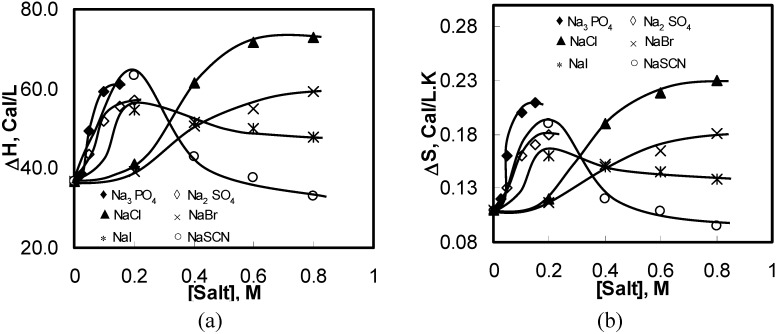
Thermodynamic properties of 1 wt% HPMC aqueous solutions of as a function of the concentration of the added salts during the heating process: (**a**) ΔH; (**b**) ΔS.

Figure 5 shows variations in the endothermic ΔH and ΔS values for the aqueous solutions of HPMC with various salts and salt concentrations. The endothermic ΔH and ΔS increased with the increasing salting-out salt concentration. All salting-out salts showed similar trends as NaCl shows. The changes in the ΔH and ΔS values for HPMC in salting-out salt solutions could be explained using the same mechanism as for the NaCl. On the other hand, the endothermic values of ΔH and ΔS for salting-in salts such as NaI and NaSCN showed a different pattern than salting-out salts. Both ΔH and ΔS increased initially with increasing salt concentration until the salt concentration reached 0.2 M. Subsequently, the quantities decreased with further increase in the salt concentration.

As stated earlier, salting-in salts are the demolishers of the oriented structure of water molecules, which enhances the intermolecular hydrogen bonding and allows denser water cages developing around the side chains of the HPMC molecules (see Figure 3(b1,b2)). As a result, higher amount of energy is required to break these water cages before any hydrophobic association between the HPMC molecules leading to the gel formation. With further increase in the salt concentration, the water cages strengthen further; some are too strong to be broken even at high temperatures. This eventually reduced the energy requirement for further hydrophobic association and gel formation causing a reduction in ΔH and ΔS values.

### 3.3. Rheological Behavior

**Figure 6:**
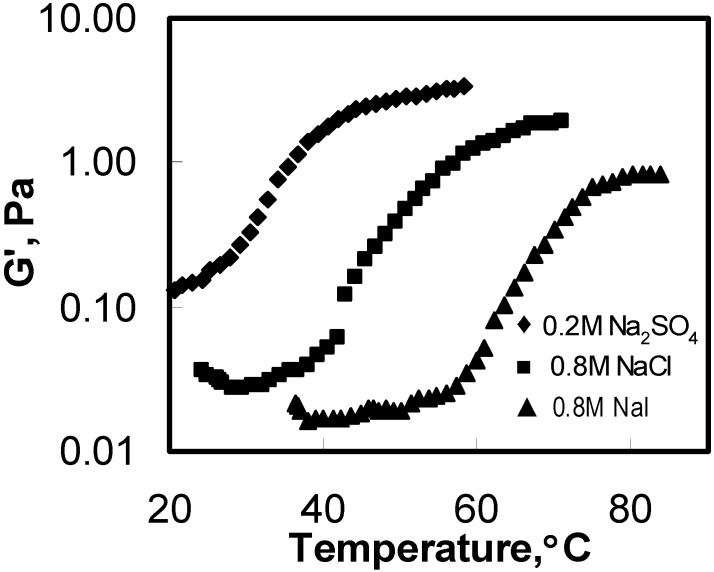
G′ as a function of temperature for the aqueous solution of 1 wt% HPMC with various salt additives measured during heating process (frequency = 1 rad/s, heating rate = 1 °C/min).

Viscoelastic characteristics of HPMC gels were studied using micro-DSC in terms of G′ and G″. The changes in G′ of HPMC upon heating as the effects of different salts additives are illustrated in Figure 6. Typical samples of 0.2 M Na_2_SO_4_, 0.8 M NaCl, and 0.8 M NaI are chosen for comparison.

The general pattern exhibited in G′ values for the samples in the presence of Na_2_SO_4_ and NaI were found to be similar to that of NaCl. The curves shifted towards lower temperature in the presence of the salting-out salt NaCl. This tendency became more pronounced in the presence of Na_2_SO_4_, a multivalent salting-out salt. In contrast to salting-out salts, the G′ curve for NaI shifted towards higher temperature, indicating salting-in effect. The final values of G′ were salt-dependent.

**Table 3:** G′ and G″ for HPMC aqueous solutions with various salts and salt concentrations measured at 70 °C, 1 rad/s frequency and 5 wt% strain.

	Aqueous solution	0.8M NaI	0.2M NaCl	0.4M NaCl	0.6M NaCl	0.8M NaCl	0.2M Na_2_SO_4_
G′ (Pa)	16.33	11.25	19.24	31.48	40.88	44.60	65.38
G″ (Pa)	2.91	2.82	4.80	3.08	3.57	11.60	18.74

As seen in Table 3, G′ increased with the addition of salting-out salts, whereas it decreased in the presence of salting-in salts. This means that the gel was strengthened in the presence of salting-out salts and weakened when salting-in salts were added in. A similar trend was reported by Cho *et al*. [35] in their study of the effects of salts on the viscosity of polyorganophosphazenes. As discussed earlier, the thermally induced gelation of HPMC solutions mainly involves hydrophobic association, which leads to a three-dimensional network. Therefore, gel strength is governed basically by the hydrophobic associations.

In the presence of salting-out salts, the number of physical junctions formed by the hydrophobic association and the strength of association went up, resulting in the increased gel strength [35]. In contrast, NaI showed a salting-in effect and enhanced the overall solubility of the HPMC chains in water, thereby causing a decrease in the gel strength. G′ values increased with the increasing NaCl concentration. Moreover, the trend correlated well with that of the ΔH. This demonstrates further that the gel strength is affected by the hydrophobic associations.

## 4. HPMC Gelation with Surfatants as Additive

### 4.1. Why Surfactants?

Amphiphilic nature of surfactants provides them with special properties to induce interactions with water-soluble polymers, especially those with hydrophobic segments/blocks. As far as ionic surfactants are concerned, reduction in surface tension and electrostatic interaction are the two main driving forces that introduce variations of aggregation patterns and phase change in aqueous solutions of water-soluble polymers [36,37].

Because of a wide range of applications of aqueous mixtures of cellulose derivatives and surfactants in pharmaceutical, cosmetic, and food industry [38,39,40], study of the thermal behavior of these mixtures has generated a considerable interest among the research community. The strong tendency of surfactants to self-aggregation induces changes in the thermal behavior of cellulose derivatives during the state change process. With priority binding to the hydrophobic parts of a carbohydrate polymer, surfactant molecules tend to aggregate around the hydrophobic segments of the polymer in an aqueous environment. This either promotes integration between the polymer chains or solubilizes the amphiphilic polymer in different modes during the state-change related processes [41,42,43].

Hoffman *et al*. [44] studied the effects of anionic surfactants such as SDS and sodium tetradecylsulfate (STS) on the gelation of hydroxyethyl cellulose (HEC) and modified HEC samples with either cationic groups (cat-HEC) or cationic and hydrophobic groups (cat-HMHEC). Kästner *et al*. [45] reported that with the addition of an oppositely charged surfactant, the modified HEC solutions showed an associative phase separation at a certain concentration of the surfactant. Resolubilization was observed with excess surfactant concentrations. The cationic and hydrophobic parts of the modified HEC interacted synergistically with anionic surfactant molecules, leading to stronger viscoelastic properties than that of cationic HEC at the same conditions. According to Evertsson and Nilsson [38], hydrophobically modified ethyl hydroxyethyl cellulose (HM-EHEC) self-associates and forms polymeric micelles in solutions. A significant rise in micro-viscosity and some reduction in micro-polarity were observed by them upon successive addition of SDS. A minor non-cooperative binding of SDS to HM-EHEC started from low concentration of SDS (<5 mM), followed by a highly cooperative binding region at SDS concentration of ≥5 mM. In general, monomeric surfactant and the composition of the formed micellar aggregation between the bound surfactant and the hydrophobic segments, both induce aligning of polymer chains as physical cross-links sites [46].

The tendency of oppositely charged surfactants and polyelectrolytes to bind together is governed by the critical aggregation concentration (CAC) of the polymer. The strong surfactant/polyelectrolyte interaction may lower the CAC value and counteract polymer solubility, resulting in gel formation [47,48]. In the case of surfactant/non-ionic polymer mixtures, significant interactions occur only after the surfactant concentration reaches its CAC value [42]. The free surfactant molecules continue to bind to the polymer through adsorption or cluster formation until the state of saturation is reached. It is believed that the non-ionic polymer will change into a polyelectrolyte-like polymer when ionic surfactant molecules are adsorbed onto the polymer via its hydrophobic tail. The electrostatic repulsions between ionic heads of the surfactant molecules tend to change the way the polymer chains align with respect to other, thereby affecting the microstructure of the corresponding gel [49,50,51,52]. As far as ionic surfactants are concerned, the anionic surfactants generally cause the viscosity to increase in comparison with the cationic surfactants due to stronger interactions in the polymer/surfactant mixture. The effect, however, varies with the chain length in a homologous series of surfactants [53,54].

In the sections below, the investigations on the effects of three anionic surfactants, namely; SDS, SDeS, SHS, and one non-ionic surfactant, Triton X-100, on thermodynamic behavior of HPMC hydrogels are discussed.

### 4.2. Gelation with SDS

**Figure 7:**
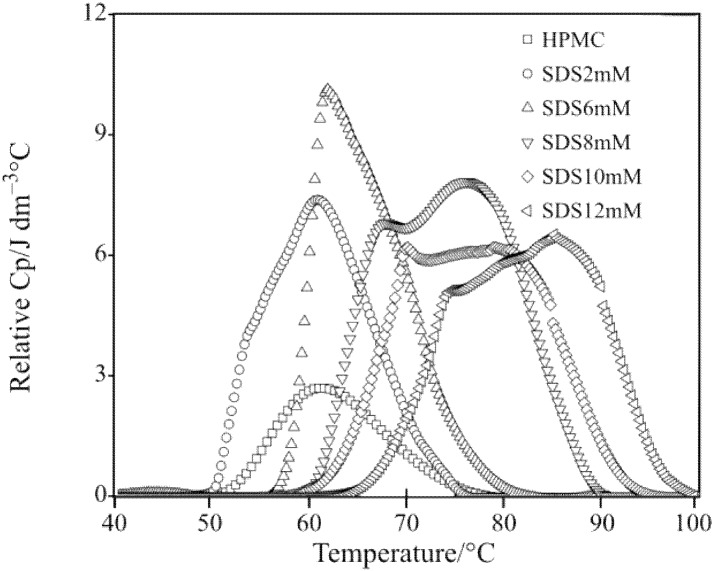
Relative heat capacity of 1.0 wt% HPMC solutions with different concentrations of sodium n-dodecyl sulfate (SDS) as a function of solution temperature.

The relative heat capacity profiles depicting thermal behavior of HPMC/SDS solutions with different SDS concentrations determined using micro-DSC with increasing solution temperature are shown in Figure 7.

In the absence of SDS, the DSC measurement for HPMC solution showed a small peak at 61 °C. However, with the addition of SDS, either the height or the position of the peak altered. As seen in Figure 7, at SDS concentration of 2 mM, the DSC profile was distinctly different from that for the pure HPMC solution. The relative heat capacity for the sol-gel transition processes significantly increased as compared with the pure HPMC solution. Up to SDS concentration of 6 mM, the peak of the corresponding curves appeared approximately at the same temperature as that for the pure HPMC solution. However, the onset of the sol-gel transition appeared to have delayed with the SDS concentration of 6 mM and higher. Based on these observations, it is very clear that the SDS concentration of 6 mM had a unique and different influence on the gelation of HPMC. At SDS concentrations higher than 6 mM, the sol-gel transition started at even higher temperatures with the shape of the peak of the corresponding curves changing from single mode to bi-mode. Each curve covered a wider range of temperature with a reduced height of the first peak.

**Figure 8:**
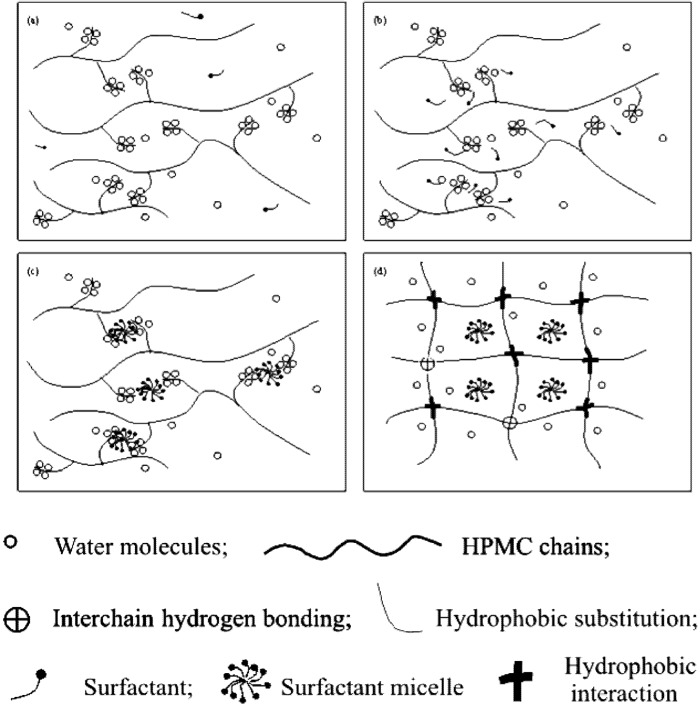
Schematic diagram showing interaction between HPMC and SDS: (**a**) SDS concentration lower than 6 mM; (**b**) SDS concentration between 6 and 8 mM; (**c**) SDS concentration higher than 8 mM; (**d**) the final network structure of HPMC gel.

Based on these DSC observations, a schematic diagram of the interaction between HPMC and SDS as well as the gelation of HPMC/SDS system was constructed and is shown in Figure 8.

Below 6 mM concentration, SDS existed as dissociative ions and no integrated units came into being (Figure 8(a)). Below HPMC concentration of 1.0 wt%, there were negligible interactions between SDS and HPMC when SDS concentration was less than 6 mM. Therefore, the presence of SDS in this concentration range did not significantly affect the gelation of HPMC; the water cages broke upon heating and the sol-gel transition took place owing only to the hydrophobic association of the HPMC chains. The *C*_p_ values only increased quantitatively due to the presence of SDS. When the concentration of SDS was higher than 6 mM, the gelation phenomenon of HPMC was affected significantly. Thus, the concentration of 6 mM for SDS can be considered as the CAC value in the presence of HPMC. After reaching the CAC value, binding of SDS to HPMC occurred either through adsorption or through cluster formation (Figure 8(b)). For a binary SDS/water system, it is well characterized that the critical micellization concentration (CMC) of SDS is around 8 mM. Since the interaction between SDS and HPMC molecules supposedly starts at 6 mM SDS concentration, the formation of SDS/HPMC complex is energetically more favorable than the formation of SDS micelles. Between the CAC (6 mM) and CMC (8 mM) values, the SDS molecules continually bind to the available sites of HPMC as either monomeric surfactants or small micelles of low aggregation number.

Above 8 mM concentration, micelles of larger aggregation number began to form around the side groups of HPMC chains (Figure 8(c)). The SDS-HPMC interaction was intermolecular in nature and it was likely that one micelle was shared by two or more HPMC molecules, creating a three-dimensional network. This binding occurred at a temperature lower than the gelation temperature and continued until the saturation of HPMC molecules with SDS as evidenced by the conductivity measurement of such a system [42]. With the continued heating, the binding and the hydrophobic association between neighboring HPMC chains progressed. The SDS units gradually moved away from the side chains of HPMC along with the breaking of the water cages. During the heating process, the breaking away of both the surfactant micelles and the water cages needed more energy. Consequently, the endothermic peaks appeared on curves at higher temperatures. As the SDS micelles and water cages were removed, the hydrophobic groups of HPMC lay exposed and the intermolecular association occurred among them, leading to the formation of new junctions for the gel network (Figure 8(d)). This delayed the phenomenon of gel formation attributed to the second peak in the thermograms; refer to Figure 7.

### 4.3. Kinetics of Gelation with SDS

The gelation kinetics of HPMC with and without SDS was studied using the DSC measurements. A concept of the degree of conversion, $\alpha_{gi}$ , was proposed for describing the progress of the gel formation process as:

(1) $$\alpha_{gi}=\frac{{\left(\Delta H\right)}_{Ti}}{\Delta H_{0}}$$

where (∆*H*)*_Ti_* is the heat released during the gel formation process at temperature *T_i_* and is calculated as $\Delta H_{i}=\int_{T_{on}}^{T_{i}}C_{Pi}dT$.

The total heat of the gelation, ∆*H_0_*, is estimated as $\Delta H_{0}=\int_{T_{off}}^{T_{on}}C_{Pi}dT$.

Correspondingly, the rate of gelation, ${\left(d\alpha_{g}/dT\right)}_{i}$ , may be calculated numerically as:

(2) $${\left(\frac{d\alpha_{g}}{dT}\right)}_{i}=\frac{{\left(dH/dT\right)}_{i}}{\Delta H_{0}}$$

where *(dH/dT)_i_* is the peak height of the thermogram at temperature *T_i_*.

**Figure 9:**
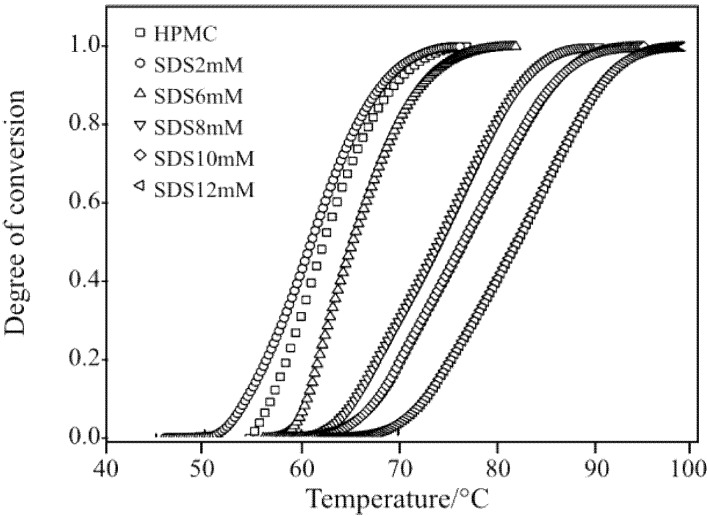
Effect of SDS on the gelation of 1.0 wt% HPMC hydrogel.

Figure 9 shows the values of the degree of conversion (determined using Equation (1) and the data presented in Figure 7) for HPMC samples with and without SDS. The sol-gel transition of the pure aqueous HPMC hydrogel started at around 55 °C. With the increasing temperature, the degree of conversion increased sharply and the gelation completed at 77 °C. With the addition of SDS at a low concentration, *i.e.*, 2 mM, the curve for the degree of conversion shifted to the left hand side with a lower slope as compared to that of HPMC solution without SDS. The sol-gel transition for aqueous HPMC/SDS (2 mM) system was observed to occur at the temperature of 51 °C. At a higher SDS concentration of 6 mM, the curve for the degree of conversion shifted to the right hand side with its slope comparable to pure HPMC solution. The sol-gel transition of the aqueous HPMC/SDS system with 6 mM SDS occurred at a higher temperature of about 58 °C. Further increase in the SDS concentration resulted in continuous shifting of the degree of conversion curve with decreasing slope.

**Figure 10:**
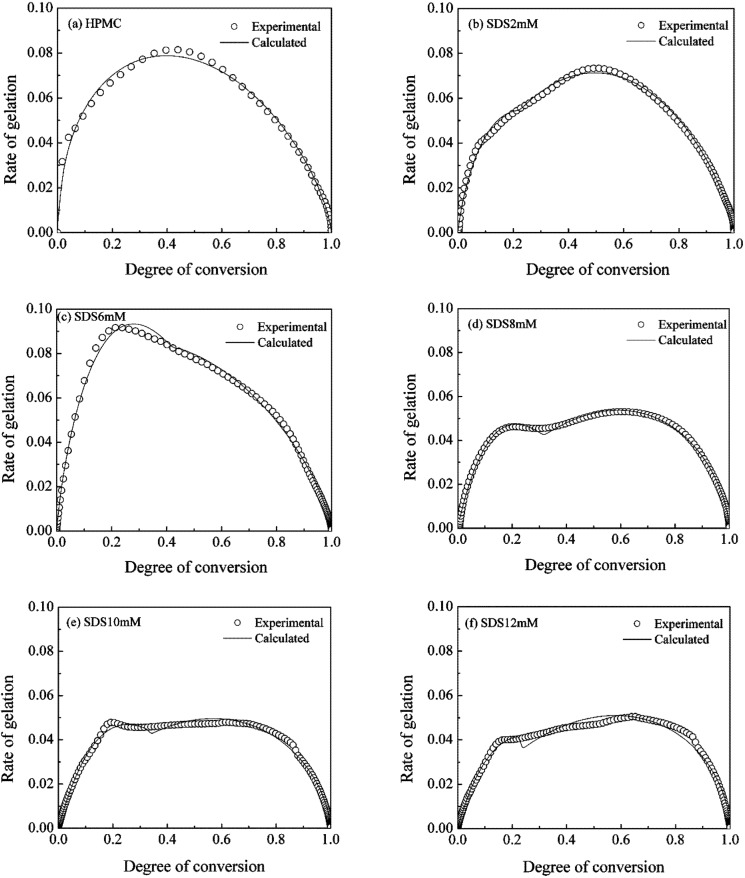
Effect of SDS on gelation kinetics for 1.0 wt% HPMC hydrogel.

The symbols in Figure 10 are the experimental data representing the rate of gelation ${\left(d\alpha_{g}/dT\right)}_{i}$ as a function of $T_{i}$ calculated using Equation (2).

A popular kinetic model from Sourour and Kamal [55]: as in Equation (3) has been used earlier to describe the isothermal gelation kinetics:

(3) $${\left(\frac{d\alpha_{g}}{dt}\right)}_{i}=\left(k_{1}+k_{2}{\alpha_{gi}}^{m}\right){\left(1-\alpha_{gi}\right)}^{n}$$

where *k_1_* and *k_2_* are temperature-dependent rate constants and *m* and *n* are empirical constants. For non-isothermal gelation process, Equation (3) may be revised as:

(4) $${\left(\frac{d\alpha_{g}}{dT}\right)}_{i}=\left(k_{1}+k_{2}{\alpha_{gi}}^{m}\right){\left(1-\alpha_{gi}\right)}^{n}$$

Based on the fact that the gelation rate is zero at the beginning of the heating process, the value of *k_1_* would have to be zero. Thus, a simplified expression for non-isothermal gelation taking place at a constant rate of heating is:

(5) $${\left(\frac{d\alpha_{g}}{dT}\right)}_{i}=k{\alpha_{gi}}^{m}{\left(1-\alpha_{gi}\right)}^{n}$$

where *k* is the rate constant for the gelation process.

Subsequently, kinetic parameters k , m and n were determined by fitting the available experimental data presented in Figure 10 to Equation (5) using nonlinear regression analysis. For the experimental data depicting the effects of SDS at all concentrations, nonlinear regression was carried out in two steps, which was based on the fact that the corresponding curves had two peaks. The values of all four parameters are tabulated in Table 4 for the corresponding curves shown in Figure 10.

**Table 4:** Kinetic parameters for non-isothermal gelation of 1.0wt% HPMC.

SDS concentration (mM)	k (min^−1^)		m		n	
	for peak 1	For peak 2	for peak 1	For peak 2	for peak 1	For peak 2
0	0.17	–	0.46	–	0.69	–
2	0.18	–	0.62	–	0.70	–
6	0.21	–	0.51	–	0.84	–
8	0.48	0.15	0.95	0.90	3.49	0.62
10	0.42	0.13	0.98	0.80	2.87	0.60
12	0.36	0.12	0.95	0.76	2.96	0.55

A reasonable mapping of the experimental data may be seen with Equation (5). Interestingly, the kinetic rate constant increased from 0.17 min^−1^ to 0.50 min^−1^ with the increasing SDS concentration from 0 to 6 mM. As discussed above, SDS existed as dissociative ions in the aqueous HPMC/SDS hydrogels and no integrates came into being in this concentration range. Such free SDS molecules would attract the water molecules, resulting in an increasing hydrophobicity due to these water molecules available for HPMC chains [56]. The sol-gel transition during the heating process was accelerated because of this enhanced hydrophobic association of HPMC. At higher SDS concentrations (≥8 mM), SDS micelles as well as HPMC/SDS complexes coexisted in the aqueous HPMC/SDS solution. Upon heating, the drifting away of SDS from the side chains of HPMC and the breaking of the water cages corresponded to the first peaks seen in Figure 10(d–f). The rate constant for this segment decreased from 0.48 to 0.36 min^−1^ with the increasing SDS concentration from 8 mM to 12 mM. With continuous heating, hydrophobic groups of the HPMC were exposed and intermolecular association occurred to form new junctions for the gel network. This process corresponded to the second peaks observed in Figure 10(d–f), and the rate constant for this process decreased from 0.15 to 0.12 min^−1^.

### 4.4. Effect of SDeS

**Figure 11:**
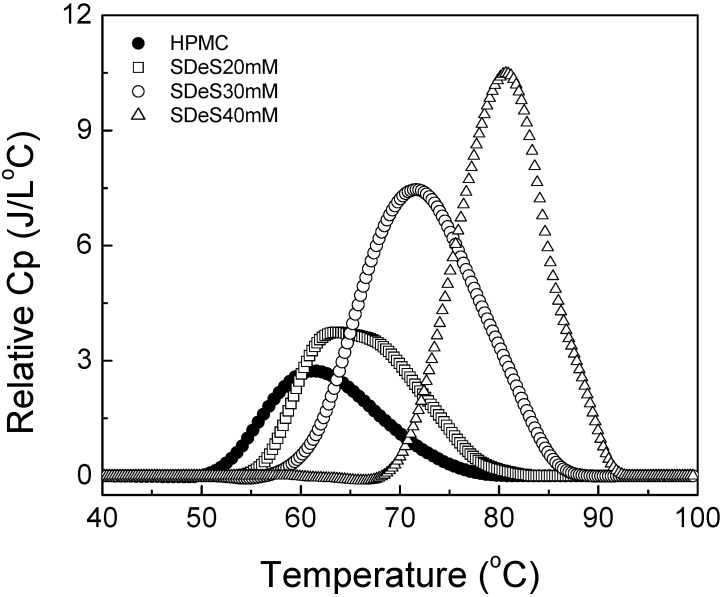
Relative heat capacity as a function of temperature for 1 wt% HPMC solutions with different concentrations of sodium n-decyl sulfate (SDeS).

Figure 11 shows thermograms for HPMC with SDeS added in. It is reported that the CMC value for SDeS in aqueous solution is 33 mM [57]. With the addition of SDeS in different concentrations, *i.e.*, 20, 30 and 40 mM, a strong “salt-in” effect in thermograms was observed and the gelation of HPMC occurred at higher temperatures. The occurrence of the highest relative *C_p_* on the thermogram shifted to a temperature of 20 °C higher. Although the shape of the thermograms had only one peak, the temperature range for the sol-gel transition became narrower with the increase in the concentration of SDeS. SDeS, being a similar surfactant to SDS with a polar head, has a propensity to bind to the hydrophobic segments of HPMC. Due to its higher concentration, the bound SDeS granted high-density electrostatic repulsive interactions, which allowed more hydrophilic surfactant shells around the HPMC resulting in a higher onset gelation temperature.

### 4.5. Effect of SHS

**Figure 12:**
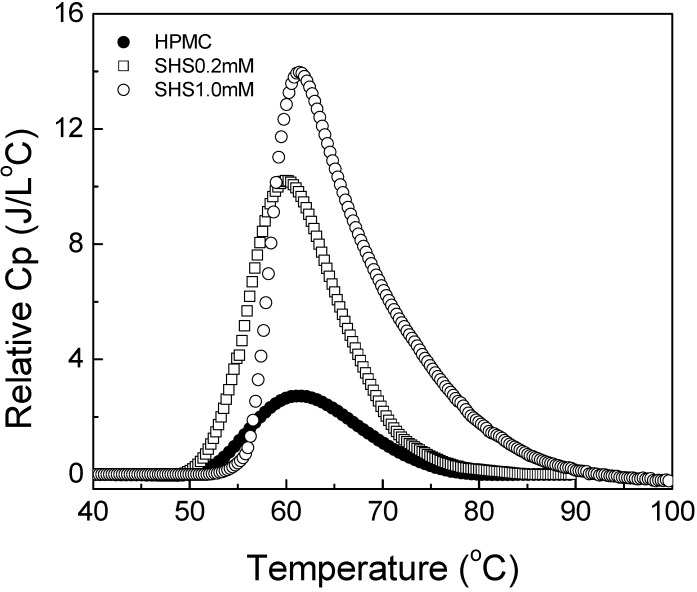
Relative heat capacity as a function of temperature for 1 wt% HPMC solutions with different concentrations of sodium n-hexadecyl sulfate (SHS).

The thermograms of HPMC in the presence of SHS are presented in Figure 12. With the addition of 0.2 mM SHS, the onset temperature for the HPMC solution did not change. The peak of the thermogram appeared at a temperature slightly lower than that for the pure HPMC solution, and a higher *C_p_* was registered. It should be noted that SHS concentration of 0.2 mM is lower than its CMC, *i.e.*, 0.45 mM of SHS in aqueous solutions [57,58]. Thus, the addition of SHS had similar influence as that of SDS at low concentrations. With the increase in SHS concentration from 0.2 mM to 1.0 mM, gelation of the HPMC solution started at a higher temperature; the behaviour similar to the gelation due to SDeS. The corresponding thermogram shifted slightly to the right side although the peak of the thermogram appeared nearly at the same temperature as that for the SHS-free HPMC solution.

### 4.6. Effect of Triton

**Figure 13:**
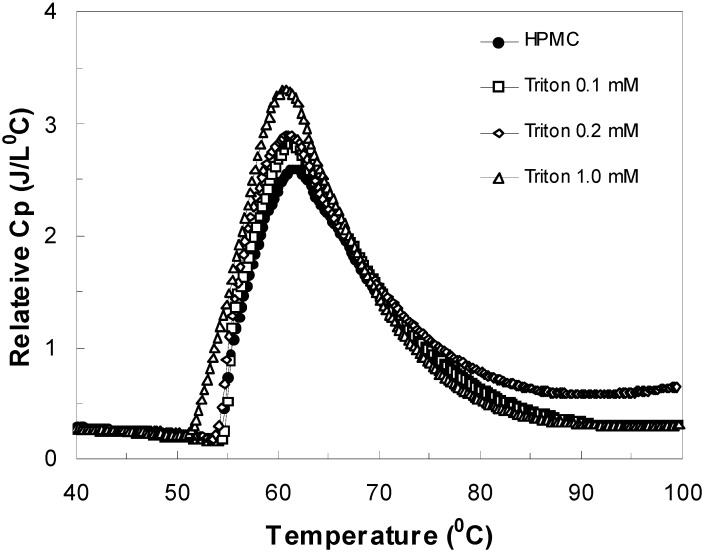
Relative heat capacity as a function of temperature for 1 wt% HPMC solutions with different concentrations of Triton.

It is known that interactions between non-ionic surfactants and neutral polymers are very weak [54,59]. Hydrophobic interaction and hydrogen bonding therefore are main interactions involved in the aggregation/dismantling of polymer/non-ionic surfactant systems [60]. As seen in Figure 13, the gelation pattern exhibited a different trend as compared to those observed with the anionic surfactants (SDS, SHS, and SDeS) when Triton X-100 was introduced into the aqueous solutions of HPMC. With the addition of Triton in different concentrations, *i.e*., 0.1, 0.2 and 1.0 mM, minor “salt-out” effect (about 1.8 °C) was observed and the HPMC gelation occurred at lower temperature. The enthalpy change in the gelation process was much less. It has been reported that the CMC value for Triton X-100 in aqueous solution is approximately 0.2 mM [61]. Any variation in the Triton concentration (below or above its CMC) did not increase the enthalpy for the sol-gel transition significantly. Thus, the similar thermal behavior as the surfactant-free HPMC solution was maintained, which essentially points out to the weaker interactions between the surfactant and the polymer. This may be attributed to the absence of dominating electrostatic association among the Triton molecules.

### 4.7. Electrostatic Effect of Surfactants

As seen in Figure 8(a) for SDS, some of the nicely integrated water cages broke partially when some of the SDS molecules get attached to hydrophobic segments of HPMC. As a result, such partially “caged” hydrophobic segments of HPMC show some polarity due to the attached polar head of the SDS molecules. The polar heads of SDS bound to the hydrophobic parts of HPMC introduce hydrophilic outer shell to solubilize HPMC chains. The increasing electrostatic repulse from the bound SDS also hinders the movement of the approaching HPMC chains in the neighborhood. In total, more energy is required to overcome these activities and to dismantle any such structure.

**Figure 14:**
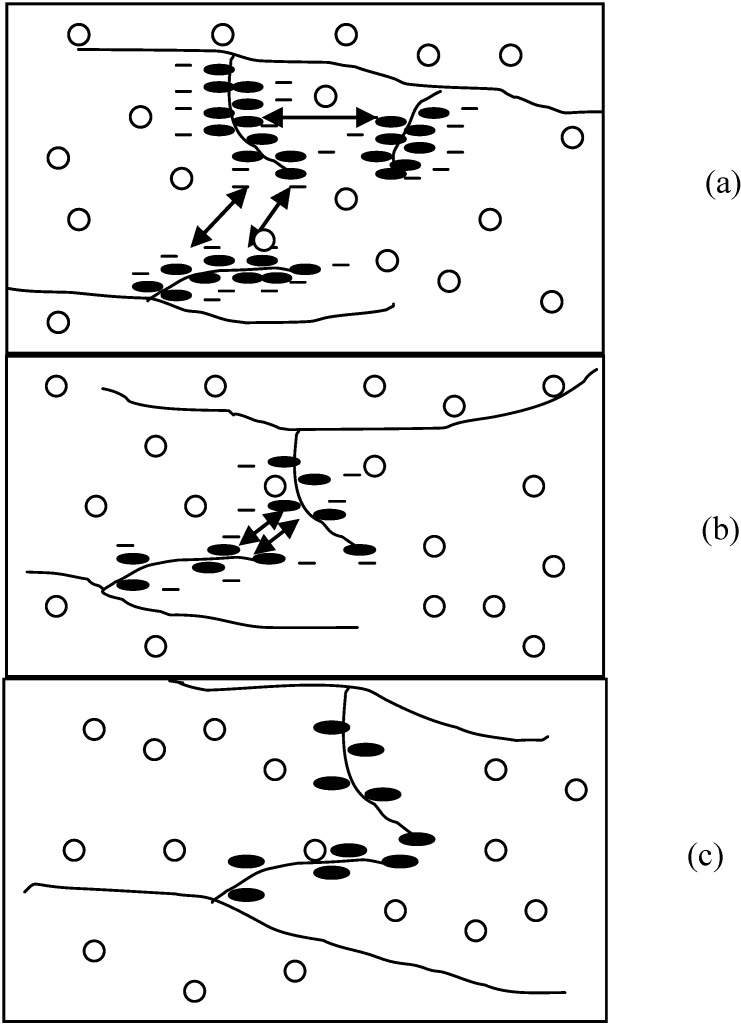
Molecular interaction between HPMC and surfactants molecules: (**a**) with SDeS; (**b**) with SHS; (**c**) with Triton X-100.

With 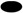 representing the polar head of surfactant molecule and 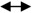 representing the electro-static repulsory, Figure 14 depicts the molecular interaction between HPMC and the surfactants.

The different strengths of electrostatic repulsion for SDeS and SHS molecules, as shown schematically in Figure 14(a,b), seemed to be the key factor in determining the behavioural difference during the thermally driven sol-gel transitions in HPMC-surfactant mixtures. The strength of the electrostatic repulsion and its variation for SDeS and SHS (much higher for SDeS) affect the processes of inducing micelle like aggregation and dismantling of the bound surfactant. This, in turn, influences the gelation, and determines the thermogram pattern and the overall enthalpy for the sol-gel transition.

Considering its non-ionic nature, the addition of Triton X-100 did not introduce any electro-statically repulsive interactions among the surfactant molecules, as shown schematically in Figure 14(c), at the time when the hydrophobic segments of HPMC were coming closer. Consequently, the energy required for dismantling any aggregation of the surfactant molecules was much less, which was also due to the lower CMC.

With the addition of surfactant, be it either anionic or non-ionic, the bound surfactant around the hydrophobic parts of HPMC will require more energy to induce alignment of the polymer chains as well as to dismantle the micellar structure. This automatically raises the energy requirement for the system; the magnitude, however, is controlled by the electrostatic repulsion and its strength.

In summary, anionic surfactants increase the energy barrier of the sol-gel transition due to their priority binding to the hydrophobic parts of HPMC, which induces polar outshells and thus hinders the free access to HPMC chains at elevated temperature. The non-ionic surfactant shows much less influence on the gelation of HPMC solution. The difference in the chemical structure of and the electrostatic interaction between the surfactant and HPMC molecules determines the thermal energy requirements for sol-gel transitions in the ternary mixtures of surfactant-HPMC-water.

## 5. Gelation of HPMC in Simulated Body Fluids

HPMC is the most commonly used hydrophilic carrier for oral-administered controlled drug release systems [62] as it displays good compression characteristics and swelling properties. Moreover, it offers a high level of drug loading as compared to MC [63]. When HPMC matrix comes in contact with water, the polymer begins to swell and forms a protective gel around the tablet content, leading to a sustained release of drugs [64]. In addition to drug delivery, thermoreversible hydrogels have been most recently used as engineering scaffolds for tissue growth [65].

It was of interest to investigate the effects of physiological buffers on the thermogelation behavior of HPMC. Thermal properties of HPMC in simulated gastric fluid (SGF) and simulated intestinal fluid (SIF) were examined. SIF, without pancreatin, was prepared using KH_2_PO_4_ and NaOH. SGF, without pepsin, was prepared using NaCl and HCl according to the specification given in the United States Pharmacopeia (25th edition). The compositions of the various buffer solutions are listed in Table 5. For the SIF buffer solutions with different pH values (5.8, 6.6, 7.4, 7.8), they were prepared using 0.05 M KH_2_PO_4_; NaOH was added to adjust pH values. For the SIF buffer solutions with different pH values but with the same buffer content (8.6 g/L), NaCl was added to keep the buffer content constant. Unless mentioned otherwise, the pH of SIF used was 7.4 and the solvent was H_2_O. The pH of SGF was 1.2, whereby the concentration of HCl was 70 mM and NaCl was added to tune the buffer content. The HPMC solutions were prepared by dissolving the polymer into respective buffer solutions.

**Table 5:** Composition of SGF and SIF solutions.

	SGF content (pH = 1.2)			SIF content (pH = 7.4)		
	4.6 g/L	9.2 g/L	18.4 g/L	8.3 g/L	16.6 g/L	33.2 g/L
NaCl (mM)	34	114	270	–	–	–
HCl (mM)	70	70	70	–	–	–
KH_2_PO_4_ (mM)	–	–	–	50	100	200
NaOH (mM)	–	–	–	38	76	152

When the number average molecular weights (Mn) of the polymers were determined by GPC, polymer sample was diluted 6 times and the solution was then filtered. The LCST (lower critical solution temperature) values for the solutions were determined at the temperatures which showed an optical transmittance of 50% during UV-vis spectrometer measurements. During rheological studies, and G′ and G″ values were examined as a function of temperature from 20 °C to 75 °C at a frequency (×) of 1 rad/s and a heating rate of 1 °C/min. Isothermal frequency sweeps (x = 0.1–100 rad/s) on HPMC solutions were also performed at 5 wt% strain.

**Figure 15:**
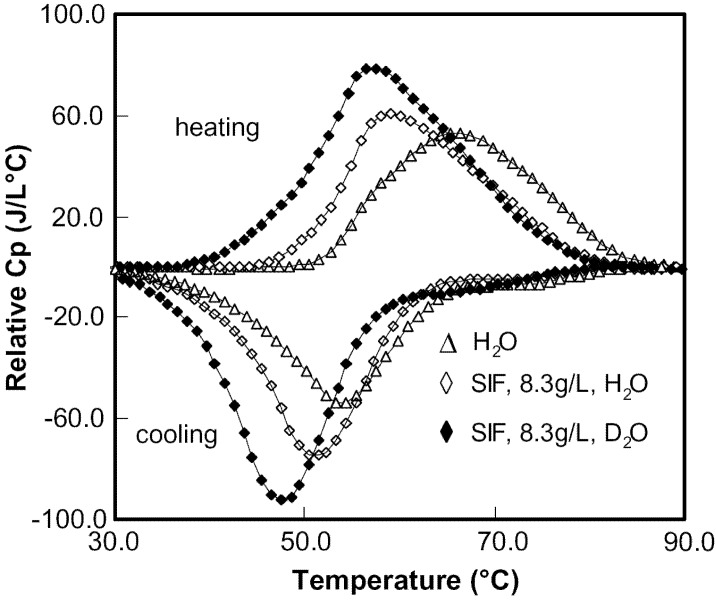
Thermal behavior of HPMC (10 wt%) in various solutions.

The thermogram of 10 wt% aqueous solution of HPMC as well as the thermograms of HPMC containing SIF (8.3 g/L) in two solvents (H_2_O and D_2_O) are presented in Figure 15. This concentration was used as a typical example because it could form strong gel as shown later. Thermograms with similar patterns were also observed for HPMC containing SGF but data are not shown in the paper to avoid repetition. In order to examine the degradation effect of SGF on the thermal gelation of HPMC, molecular weight of HPMC in SGF (4.6 g/L) before and after micro-DSC experiment were measured using GPC. The Mn of HPMC measured before and after the micro-DSC experiment was 8.1 K and 8.3 K, respectively, indicating that there was no degradation of HPMC in SGF during the experiments.

The curves for HPMC in SIF were found similar to those in aqueous solution. However, T_max_, defined as the sol-gel transition temperature [66], shifted to a lower temperature. For example, T_max_ for the endothermic curve was 66.0 °C in aqueous solution while it was 59.2 °C in the presence of SIF (8.3 g/L). During the heating process, a broad endothermic peak was observed while two exothermic peaks (a broad exothermic peak and a small shoulder) were present during the subsequent cooling process. The similarities between the patterns of the thermograms for HPMC in the presence of SIF and in aqueous solutions indicate that they share the same mechanisms as described previously [67,68]. It has been well known that the formation of intermolecular hydrogen bonding between hydroxyl groups of HPMC chains and water molecules as well as water cages surrounding hydrophobic clusters of HPMC chains such as methoxyl substituted and relatively less hydrophobic hydroxypropyl substituted regions makes HPMC soluble at low temperature [67]. The concept of water cages may be defined as the formation of enhanced water–water hydrogen bonding in the hydrophobic hydration shell [31]. The intermolecular hydrogen bonding is gradually weakened with increasing temperatures. Upon heating to a temperature to the onset of the endothermic peak (T_onset_), the water cages start to deform and break to expose the hydrophobic substitutions to the aqueous environment. The exposed hydrophobic substitutions form hydrophobic aggregation domains via hydrophobic-hydrophobic interactions. These domains are connected through hydrophobic groups, yielding a three-dimensional physical network of HPMC chains.

It is worth noting that the endothermic peak (Figure 15) and midpoint temperature (Table 6 and Table 7) shifted to a lower temperature, as compared with the aqueous solution of HPMC, in the presence of SIF (8.3 g/L) and SGF (4.6 g/L). This indicated that the sol-gel transition was promoted and occurred early due to the added buffer. To interpret the buffer effect on the sol-gel transition in this study, it is important to consider the effect of salt on the water structure.

As shown in Table 5, the compositions of SIF contain OH^−^ and H_2_PO_4_^−^ while those of SGF contain Cl^−^, which are water structure makers or salting-out anions according to Hofmeister series. In their presence, the adjacent water molecules were polarized and rearranged in the electric field of the ion [22]. Viscosity B coefficient is always used as an empirical constant representing ion-water interactions. Viscosity B coefficients of OH^−^ and H_2_PO_4_^−^ are 0.112 L mol^−1^ and 0.34 L mol^−1^, respectively [33]. Ions with positive viscosity B coefficient values are strong structure makers for water [22]. The structure makers have a tendency to attract water molecules around them, facilitating the sol-gel transition. This salting-out effect could be attributed to two processes [15,18,26,69]. One is that the anions competed for water molecules and the water molecules were rearranged in the electric field of the anion. As such, less free water molecules were available to solvate polymers. The intermolecular hydrogen bonding was therefore weakened and easily disrupted. Another one is that the surface tension of the water cages surrounding the hydrophobic clusters increased due to the salting-out effects of OH^−^ and H_2_PO_4_^−^, causing depletion in the strength and number of water cages and resulting in enhanced hydrophobic association at low temperatures.

**Table 6:** Thermal characteristics of HPMC thermograms for various HPMC solutions.

HPMC weight concentration	Aqueous solution			SGF solution (4.6 g/L)			SIF solution (8.3 g/L)		
	1%	5%	10%	1%	5%	10%	1%	5%	10%
Endothermic enthalpy changes (KJ/L)	0.08	0.47	1.0	0.09	0.52	1.09	0.09	0.52	1.05
Endothermic entropy changes (J/L·K)	0.23	1.39	2.95	0.24	1.53	3.21	0.24	1.56	3.14
Midpoint temperature on heating (°C) ^*^	60.6	58.2	58.0	58.5	56.5	56.8	55.3	52.3	52.5

^*^ the value of midpoint temperature on heating is defined as the average value of onset temperature (the starting temperature of the endothermic peak) and the peak temperature (the temperature at which Cp reaches the maximum).

**Table 7:** Thermal characteristics of HPMC thermograms for 10 wt% HPMC solutions with various SGF and SIF contents.

	Aqueous solution	SGF contents (g/L)			SIF contents (g/L)		
		4.6	9.2	18.4	8.3	16.6	33.2
Endothermic enthalpy changes (KJ/L)	1.0	1.09	1.35	1.75	1.05	1.38	1.72
Endothermic entropy changes (J/L·K)	2.95	3.21	4.01	5.26	3.14	4.19	5.32
Midpoint temperature on heating (°C)	58.0	56.8	53.4	47.5	52.5	47.4	38.8

The broad exothermic peak and the smaller shoulder observed during the cooling process indicated that gel-sol transition proceeded in two successive transitions. Similar trends in the thermograms of HPMC and MC in aqueous solutions have been reported by Hirrien *et al*. [70], yet their interpretations are different. The exact mechanisms cannot be obtained with thermograms alone, which will be elucidated via the viscoelastic study during the cooling process later. In the presence of SIF, the gel-sol transition was deferred during the cooling process. This is due to the competition for the free water molecules by salting-out salts.

### 5.1. Effect of Basic Solvent

**Figure 16:**
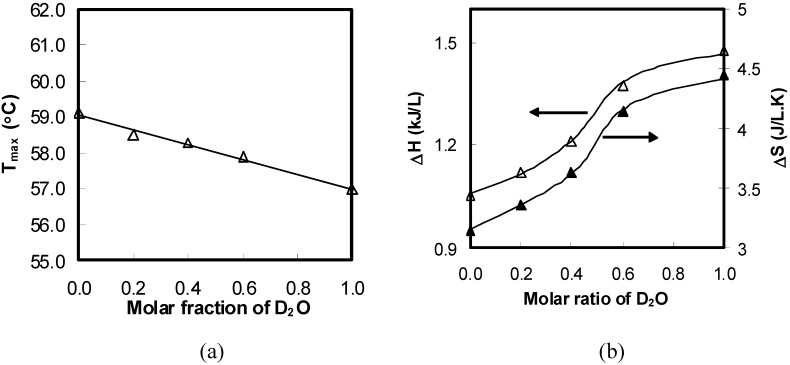
Thermodynamic properties of HPMC solutions (10 wt%, SIF 8.3 g/L) as a junction of molar fractions of D_2_O: (**a**) T_max_; (**b**) ΔH and ΔS.

Although a fairly detailed picture of hydrophobic association involved in the thermogelation has been obtained, the effect of the interchain hydrogen bonding involved in the thermogelation of HPMC is not clear yet. Since Deuterium substitution is a very useful method to investigate the properties of hydrogen bonding interactions, this effect was studied using various molar ratios of D_2_O and H_2_O. As seen in Figure 15, the patterns of the thermograms of HPMC solutions containing SIF in both solvents are very similar. However, the T_max_ in D_2_O moved to a lower temperature. More interestingly, the T_max_ has approximately a linear relationship with the mole fraction of D_2_O (see Figure 16a).

These observations are in contrast to the cases of poly(N-isopropylacrylamide) (PNIPAAm) studied by Kujawa and Winnik [71], where the T_max_ value with D_2_O is higher by 2 °C compared to that with H_2_O. This increase of T_max_ for PNIPAAm can be explained by considering that the deuterium bonding in D_2_O is about 5 wt% stronger than the hydrogen bonding in H_2_O, leading to an increase in the T_max_ [18,71]. Additionally, polymer chains are more extended in D_2_O.

The opposite results obtained in our investigations for HPMC with and without SIF indicated that interchain hydrogen bonding was involved in the gelation process, leading to a salting-out effect. In the case of HPMC, the decrease of T_max_ in D_2_O was assumed to be due to the interchain hydrogen bonding rather than the combined effects of the interchain hydrogen bonding and enhanced hydrophobic interactions. This is because a non-linear change in the T_max_ with D_2_O content instead of a linear change will be observed if D_2_O has more than two types of complex interactions with HPMC [72]. This trend is consistent with the findings reported by Winnik [73] for hydroxypropyl cellulose (HPC).

In addition to T_max_, ΔH and ΔS with various D_2_O content were extracted from the thermograms and the results are illustrated in Figure 16b. It was interesting to find that these thermodynamic parameters exhibited a significant dependence on D_2_O content and followed sigmoidal shape with the variations in D_2_O content. Three distinct regions were observed. When the molar ratio is below 0.4, ΔH and ΔS increased slightly. They underwent a rapid increase in the region where 0.4 < molar ratio < 0.6, and then gradually reached a plateau. To interpret this trend, it is necessary to investigate the thermodynamic parameters of HPMC containing SIF in H_2_O. There are several processes that might consume or release heat during thermogelation. For instance, heat was consumed to break intermolecular hydrogen bonding and water cages as well as for hydrophobic association. However, the formation of interchain hydrogen bonding is an exothermic process. It has been extensively studied and demonstrated that the positive ΔH (endothermic peak) was mainly related to the destruction of water cages around hydrophobic clusters of the polymer chains [20,74,75]. Therefore, ΔS must be positive at a given temperature to meet the requirement of ΔG = ΔH − TΔS < 0, where ΔH > 0. The ΔS caused by the formation of gel network is negative resulting from the reduction in the flexibility of polymer chains. In addition, water molecules get trapped into these gel networks, leading to negative ΔS. Such inconsistency can be resolved by consideration for smaller molecules such as water molecules; these are smaller in size but large in numbers. The water molecules involved in hydrogen bonding and water cages are relatively ordered at low temperature and become disordered upon destruction at high temperatures. More importantly, this positive ΔS value is greater than that of the negative ones to make the total ΔS value positive. This phenomenon is in line with those reported by many other researchers [20,67].

**Figure 17:**
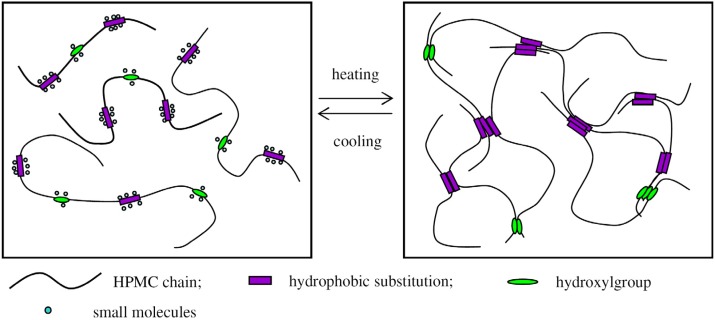
Schematic structures of sol-gel transition of HPMC aqueous solutions with buffer.

In D_2_O, it was expected that more heat be consumed to break the strengthened intermolecular hydrogen bonding. On the other hand, this is compensated by the formation of interchain hydrogen bonding because the latter is prevalent in D_2_O. The increase of ΔH and ΔS in D_2_O compared to those in H_2_O can only be explained by consideration of the small molecules including water molecules and ions surrounding hydroxyl groups of the HPMC chains (Figure 17(a)). Similar results have been reported by Weng *et al*. [12] where small molecules acted as an overcoat surrounding a cellulose chain. In our cases, the small molecules served as a shell to the hydroxyl groups at low temperatures, preventing the formation of interchain hydrogen bonding. This is very similar to the water cages surrounding hydrophobic clusters. The shells were disturbed and broken to expose hydroxyl groups at raised temperatures, leading to the formation of interchain hydrogen bonding (Figure 17(b)). It may be therefore suggested that both the interchain hydrogen bonding and the hydrophobic interactions are concerned with the gelation of HPMC, while the later plays a more important role in the gelation.

The junction zones also contain two parts: association of hydroxyl groups and hydrophobic clusters as illustrated in Figure 17(b). This conclusion is in agreement with the studies performed on MC [76].

Now, the three regions trend of ΔH with various D_2_O contents (Figure 16(b)) can be explained in terms of the strength of the shell for the hydroxyl groups. The first region indicated that the strength of the shell increased slowly, where more heat was consumed to break the shell to induce association of hydroxyl groups of HPMC chains. The second region suggested that the strength of the shell increased rapidly, where much more heat was needed. When the molar ratio of D_2_O was higher than 0.6, the absorbed heat gradually reached a plateau. This indicated that the rate of increase in the shell strength decreased gradually approaching zero. It is interesting to note that the same trend was seen for the ΔS. This further confirmed that the changes in enthalpy and entropy were related to the strength of the shell.

### 5.2. Influence of Buffer Content

**Figure 18:**
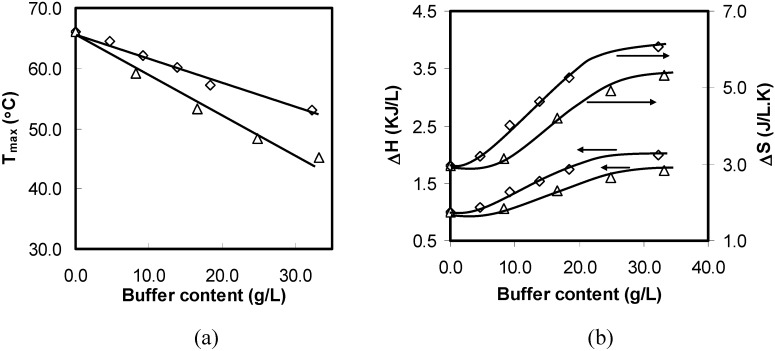
Thermodynamic properties of 10 wt% HPMC solutions (SGF ◊; SIF ∆) as a function of buffer content: (**a**) T_max_ ; (**b**) ΔH and ΔS.

With the increase in either SGF or SIF content, the DSC curves became broader (data not shown) and the T_max_ shifted nearly in a linear manner to lower values with the two curves fitted with their slopes as −0.42 °C g^−1^ L and −0.63 °C g^−1^ L, respectively; refer to Figure 18(a).

The more pronounced salting-out effect with increasing buffer content is because of the fact that buffers with higher concentrations can attract more free water molecules around the salt ions, resulting in fewer free water molecules available around the hydrophobic clusters and hydroxyl groups. The water cages around the hydrophobic clusters and the shell around the hydroxyl groups were further weakened with the increasing buffer content upon heating. Similarly, the re-formation of cage structure around the methyl groups was further deferred with high buffer content during the cooling process (data not shown). The T_max_ curve of the SIF has a higher negative slope, indicating that it has a stronger salting-out effect as compared to that of the SGF. This result can be explained by the difference in the salting out capacity of anions since cations have much less effect on the thermogelation as compared to anions. The value of the viscosity B coefficient of Cl^−^ is −0.005 L mol^−1^, which is less than those of OH^−^ and H_2_PO_4_^−^ [33]. Anions with higher viscosity B coefficient values tend to attract water molecules from polymers, water cages and shells more strongly. Therefore, salting-out effect is more pronounced in the presence of SIF.

The effects of buffer content on ΔH and ΔS are illustrated in Figure 18b. It was observed that all curves are of sigmoidal shape. The plots can be divided into three buffer content regions. For SIF content below 8.3 g/L and above 24.9 g/L, ΔH and ΔS exhibit a slight increase with the increasing SIF content. However, these quantities undergo a sharp increase in the region ranging from 8.3 g/L to 24.9 g/L. A similar trend has also been reported by Alexandridis and Holzwarth [20] for pluronics, which showed that ΔH reached a plateau at higher salt concentrations. We also showed that some heat was consumed to break the shell around the hydroxyl groups of the polymer chains. However, endothermic heat was mainly attributed to the destruction of water cages around hydrophobic clusters of the polymer chains [20,66]. In addition, the strength of the water cages reduced with the increasing buffer content. Therefore, such trends can only be explained in terms of the total number of hydrogen bondings in the water cages. This can be explained as follows. Firstly, because the distribution of methoxyl and hydroxypropyl along HPMC chains is not homogenous, it may contain trisubstituted, disubsitituted and monosubstituted units. The thermogelation of HPMC was mainly attributed to the hydrophobic interactions of trimethoxyl substitutions [70]. Secondly, lower-methoxyl substituted units and less hydrophobic substitutions such as hydroxypropyl groups become more hydrophobic in the presence of SGF and SIF due to the salting-out effects. Hence, the total number of higher hydrophobic substitutes increased. On the other hand, more new water cages are formed around these less substituted units at lower temperatures. Therefore, more energy will be needed to break these new water cages to induce hydrophobic association in addition to the highly substituted units. The sigmoid trend in Figure 18b can be interpreted in the following ways. The number of newly formed water cages increased slightly at low buffer content. It is possible to assume that salts at low concentrations mainly compete for free water molecules in bulk water instead of those in polymer–water interface. However, as the buffer content continued to increase and reached 24.9 g/L, there are enough anions to compete for a lot of water molecules in the polymer-water interface and create more hydrophobic substitutions, resulting in a rapid increase in the number of newly formed water cages. Furthermore, the number of newly formed water cages gradually reached saturation when the SIF content was above 24.9 g/L, resulting in a slight increase in ΔH. The similar observation for ΔS further confirmed that ΔH was mainly related with the number of newly formed water cages. This is because ΔS was also mainly attributed to the disruption of water cages as elaborated earlier in this study. It is also noted that the ΔH and ΔS values in the case of SGF were a little higher than those for SIF.

### 5.3. Influence of Solution pH

The effect of pH on the thermogelation is of interest since orally administrated drug loaded hydrogels are exposed to SGF (pH between 1.0 and 2.5) and followed by SIF (pH between 5.5 and 7.9) [77]. As shown in Table 8, upon heating, the T_max_ decreased slightly with increasing pH. This was caused by the slight increase in NaOH concentration because NaOH adjusted the pH. In the case of SIF with equal salt content, the similar trend was also observed. This was because the salting-out ability of OH^–^ is more pronounced than that of Cl^−^. The corresponding ΔH and ΔS of HPMC in buffers with different pH values are illustrated in Table 6 and Table 7 respectively. They all increased slightly with increasing pH, indicating that they exhibited weak pH dependence. Unlike polyelectrolytes, non-ionic polymers such as HPMC will not ionize by changing the pH. Hence, the thermal properties were not affected greatly by the pH. This finding is in agreement with some reported studies [29].

**Table 8:** Peak temperatures (T_max_) of HPMC thermograms for SIF solutions with different pH.

Parameter	SIF buffer with different pH				SIF buffer with constant buffer content (8.6 g/L)			
pH	5.8 (6.9 g/L)	6.6 (7.5 g/L)	7.4 (8.3 g/L)	7.8 (8.6 g/L)	5.8	6.6	7.4	7.8
T_max_ on heating, °C	61.69	60.90	59.17	58.98	60.73	60.54	59.05	58.98
T_max_ on cooling, °C	51.24	51.15	49.87	49.78	50.54	50.35	49.75	49.78

### 5.4. Influence of Polymer Concentration

In addition to the parameters mentioned earlier, the effect of polymer concentration on thermal behavior was examined (data not shown). The influence of polymer concentration ranging from 1 wt% to 10 wt% on the T_max_ was not significant, indicating that the gelation process was a temperature driven process rather than only driven by the heat input [78]. On the other hand, ΔH and ΔS were found to be linearly proportional to the polymer weight concentration, suggesting that more HPMC chains were involved in the gelation. Similar results are also found for HPMC in the absence of buffer.

### 5.5. Changes in Light Transmittance

**Figure 19:**
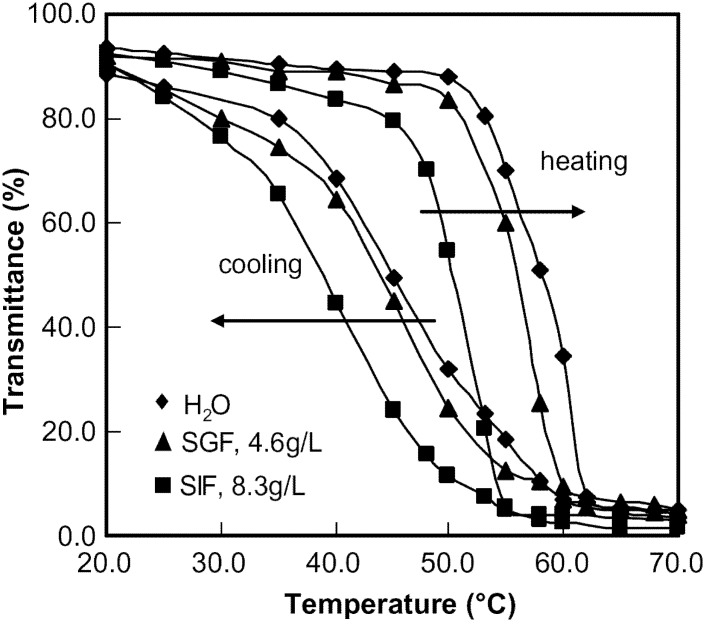
Light transmittance of HPMC solutions (10 wt%) in various solutions as a function of temperature.

Transmittance changes for HPMC aqueous and buffer solutions as a function of temperature are presented in Figure 19. The solutions were transparent below the T_onset_ but became turbid at temperatures (LCST) between the T_onset_ and T_max_ measured by the micro-DSC. During cooling, the solution became transparent at low temperatures. However, an obvious hysteresis was observed during the cooling process. The results are consistent with those obtained using the micro-DSC. It should be mentioned that the LCST was relatively independent of concentration in the studied range. However, the LCST of diluted HPMC solution (3 wt% and less) was found to increase by a few degrees. For instance, the LCST of HPMC solution (1 wt% in DI water) was 69.0 °C. The reason for this is that the polymer aggregates are slow to aggregate to a size that can be detected by the UV-vis at low concentrations. The gelation behavior induced by temperature is known as the sol-gel transition with a LCST. The LCST moved to a lower temperature in the presence of SGF and SIF.

The turbidity was attributed to the Rayleigh scattering from junctions formed in the gelation process. LCST is always considered as an indication of phase separation [79]. The relationship between gelation and phase separation for MC and HPMC has been elaborated by other researchers in their studies [80,81,82]. However, there are different opinions about the nature of the phase separation and gelation. For instance, Kobayashi [80] showed that the gelation was accompanied by liquid–liquid phase separation, as evidenced by the turbidity changes. Takahashi *et al*. [82] suggested a concurrence of phase separation and gelation. They also pointed out the possibility of gel-gel phase separation with different polymer concentrations at higher temperatures. Many other researchers proposed that the turbidity was caused by the hydrophobic interactions of the methoxyl groups on the polymer chains [68]. In our investigation, no precipitation was observed up to 75 °C. It is therefore suggested that the turbidity is more likely be due to micro-phase separation induced by hydrophobic association and hydroxyl group association. The microphase separation would in turn cause gelation rather than macro-phase separation because there is no precipitation. This phenomenon is in agreement with an observation made by Li *et al*. [66] in their studies about the formation of MC gel or microgel when the temperature was above the LCST.

### 5.6. Viscoelastic Studies

Viscoelastic properties of HPMC solutions were investigated to further understand the gelation mechanism. SIF was chosen as a typical example since HPMC in SGF and in SIF exhibited similar behavior. Additionally, changing pH did not generate significant difference on the properties of HPMC, as evidenced earlier. The concentration dependence of the quasi-equilibrium modulus $G_{E}$ was examined at a gelling temperature of 68 °C for HPMC solutions in the presence of SIF, where $G_{E}$ was defined as the storage modulus ( $G^{\prime }$ ) at the frequency of 0.1 rad/s. A scaling relation $G_{E}=\chi^{\kappa }$ has been widely used to characterize the gel state, where χ is the relative distance of a variable such as concentration or temperature from the sol–gel transition point, and κ is the exponent [83]. In this study, χ was defined as concentration instead of relative distance of concentration because the critical concentration for sol-gel transition at this temperature is difficult to obtain, as pointed out by Li [84] in his work.

**Figure 20:**
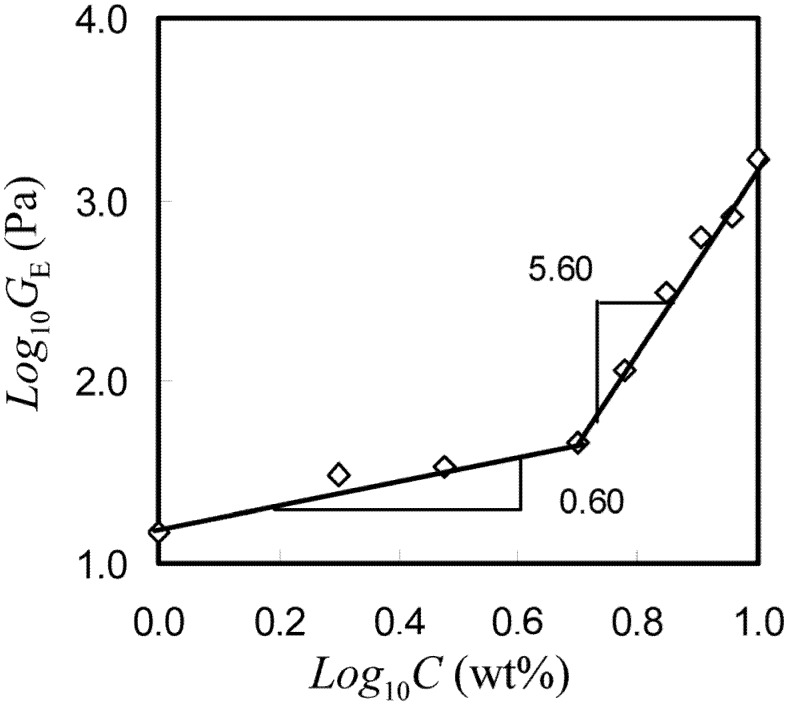
Concentration dependence of $G_{E}$ for HPMC in the presence of SIF (8.3 g/L) (frequency x = 0.1 rad/s, strain amplitude c = 5 wt% and 68 °C).

As seen in Figure 20, the slopes of the two straight-line segments were 0.6 and 5.6, respectively. The results suggested that the gels formed at concentrations below 5 wt% were weak gels while those formed above 5 wt% were strong gels. It should be noted that the definition of weak (5 wt%) was based on the scaling relations found from equation of $G_{E}=\chi^{\kappa }$. Similar results can be found in studies elsewhere, suggesting that the multi-scaling laws for the gelation of MC were due to its heterogeneous gelation network [84,85].

**Figure 21:**
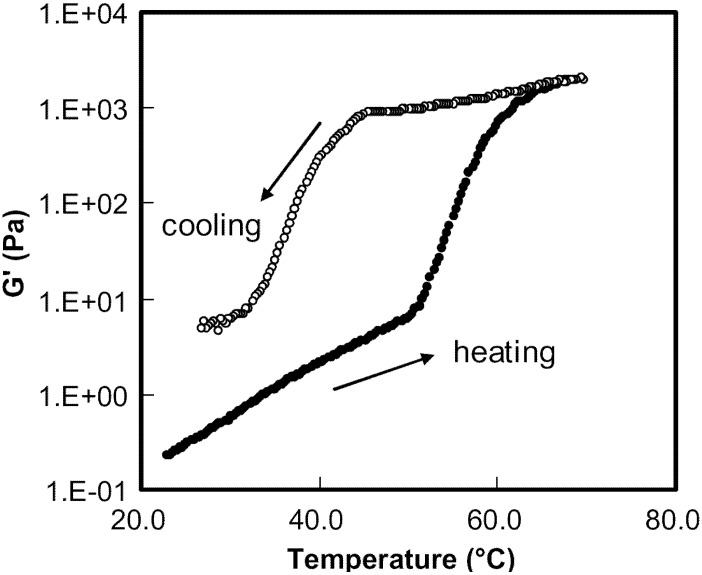
Dynamic storage modulus $G^{\prime }$ of HPMC (10 wt%) in the presence of SIF (8.3 g/L) at a frequency x = 1 rad/s and a strain amplitude c = 5 wt%.

The temperature dependence of $G^{\prime }$ for HPMC (10 wt%) in the presence of SIF (8.3 g/L) was also studied (Figure 21). It was found that the gelation proceeded in two stages. $G^{\prime }$ increased slightly with increasing temperature up to about 51 °C. This may be attributed to the entanglements of HPMC chains. Upon further heating, $G^{\prime }$ increased sharply before attaining a near plateau at 69.0 °C (1 wt% in DI water).

Rheological measurements showed that $G_{E}$ at the gel state had different scaling relations to the polymer concentration. Moreover, $G_{E}$ was affected by buffer content. Thus, it is possible to tailor the gel elasticity by varying the buffer content in addition to the polymer concentration. The temperature dependence of the viscoelastic behavior was in well agreement with the observed thermal behavior.

It was observed that the rapid increase of $G^{\prime }$ was related to the temperature region between T_onset_ and T_max_ measured by micro-DSC. The reason for such a rapid increase in $G^{\prime }$ at temperatures greater than the T_onset_ is the kinetics of the sol-gel transition, where heat was absorbed to break the water cages and the shell surrounding the hydrophobic clusters and hydroxyl groups, respectively. This was followed by hydrophobic association and hydroxyl groups association as illustrated in Figure 17. The rapid increase in $G^{\prime }$ for HPMC gel was caused by the development of a network of junction zones of hydrophobic association and hydroxyl groups association. Finally, $G^{\prime }$ reached a plateau when the formation of the gel network was mostly completed. During subsequent cooling, $G^{\prime }$ decreased slowly in contrast to the rapid increase of $G^{\prime }$ in the same temperature zone upon heating. The initial slow reduction in $G^{\prime }$ was due to the gradual weakening of the network, described by the smaller shoulder at higher temperatures. The subsequent rapid decrease in $G^{\prime }$ corresponded to the large amount of heat released by the formation of the water cages, shells and intermolecular hydrogen bonding during the dissociation of the gel network. The pattern of $G^{\prime }$ upon heating is very similar to that in the absence of buffer (data not shown). However, $G^{\prime }$ decreased gradually during the whole cooling process in the absence of buffer. The reason for the gel in aqueous solution for not able to retain its strength is because the gel network is easily weakened in the presence of hydroxypropyl groups even at higher temperatures. In the presence of SIF, the dissociation of gel structure as well as the re-formation of intermolecular hydrogen bonding, water cages and shells were deferred in the cooling process. This is due to the competition for the free water molecules by salting-out salts. G’ curve further moves to a lower temperature upon heating with the increasing SIF contents (data not shown). The salting out effect is consistent with those measured by micro-DSC.

**Figure 22:**
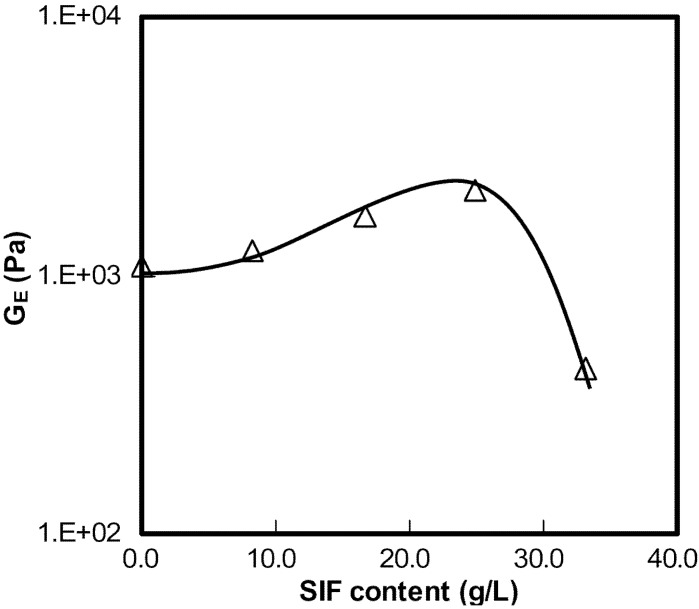
$G_{E}$ of HPMC solutions as a function of SIF content (frequency x = 0.1 rad/s, strain amplitude c = 5 wt% and 68 °C).

To compare the viscoelastic properties of the polymers in different SIF contained solutions, frequency dependence for HPMC (10 wt%) was conducted at 65 °C. $G^{\prime }$ was much greater than $G^{\prime \prime }$ (loss modulus) and showed a weak dependence over the whole frequency range. This behavior is typical for a gel. $G_{E}$ values are illustrated in Figure 22. It was found that the gel elasticity (*i.e.*, $G_{E}$ ) increased slightly with the initial increase in SIF content. This is because the presence of salting-out salts led to a stronger hydrophobic association and hydroxyl group association. This result is in good agreement with that reported by Sarkar [81]. It should be noted that $G_{E}$ increased rapidly when SIF content increased from 8.3 g/L to 24.9 g/L. This trend is similar to those for the ΔH and ΔS as stated earlier and can be explained by the cross-linking density of the physical network.

The total number of higher hydrophobic substitutes increased with increasing SIF content in a sigmoidal manner, resulting in a similar trend in the changes of the crosslinking density. However, $G_{E}$ decreased significantly when SIF content further increased to 33.2 g/L. This was caused by the macroscopic phase separation induced by this high salinity.

## 6. HPMC Tablets and Drug Delivery

Among various dosage forms in medicine, tablets still account for 80% of the drug delivery systems administered to human due to ease of manufacture, convenience of dosing and stability as compared with liquid and semi-solid dosage patterns [86]. For the dosage of an active ingredient under 30%, the direct compression to form tablets is used widely in pharmaceutical industry for ease of fabrication and robustness to handling environment [87].

Various parameters influence the drug releasing profile of HPMC tablets. Porosity of the formed tablets, different particle sizes and distribution of HPMC powder and physicochemical properties of various grades of HPMC are some of the critical factors that influence the behavior of a drug-loaded HPMC tablet in body fluids [88,89,90,91]. Many studies have been focused on swelling process for cellulose-based materials in various media. Some focused on kinetics of the swelling process and drug release profile. Kinetic studies often described the cumulative drug releasing profile as a power function of time [92,93]. The power law model, however, is too simple to express the detailed drug release profile of HPMC [94]. With further developments, more detailed models were proposed with consideration for diffusion of water and drug, and the moving boundary and swelling of HPMC system [95,96,97]. However, there is no comprehensive study on the bio-fluid uptake and disintegration of HMPC tablets with time in those fluid media, which is important to find out the key factors and mechanisms that drive and control the tablet wetting as well as the drug release processes.

For this purpose, tablets of three grades of HPMC with different properties were fabricated. To begin with, HPMC-A, -B, and -C in their powder form were dehydrated in vacuum at 60 °C for 24 h. The tablets were prepared using the direct-compression method without any extra additives or binders. The forming condition was set to 1-min compression under 400 kg/cm^2^ pressure. The average weight of the formed tablets was 1.3 g. The tablets were of cylindrical shape with their average diameter and height measured at 18 mm and 5–8 mm respectively. The tablets were stored all the time in a dry box before use.

Upon taking 10 mg of pre-dried HPMC powders A, B, C each time and sealing the sample in the standard aluminum sample pan, T_g_ of the three samples was measured using the TA modulated DSC 2920 in the range of 20–220 °C with the ramp rate of 10 °C/min under N_2_ environment maintained at flow rate of 50 mL/min.

### 6.1. Fluid Uptake and Swelling Measurements

Three full-sized tablets of each of HMPC-A, -B and -C were weighed accurately before being placed individually in a porous paper bag. The bag was used to facilitate handling of the tablets as and when required and simultaneously insure that the solvent could diffuse without any obstruction during the swelling process. A 50 mL of deionized water, SGF, and SIF were loaded in separate plastic tubes with a properly marked scale line. One set of media was used to soak one grade of HPMC tablets. Three rounds of experiments were carried out for each of HPMC-A, -B and -C tablets. All solutions were maintained at the near body temperature of 37 °C. In order to assess the water uptake at various time intervals, the swollen tablets were taken out quickly, had their surfaces wiped using a tissue paper and weighed immediately. The swelling ratios were calculated using Equation (6) as below:

(6) $$S_{r}=\frac{W_{t}-W_{t0}}{W_{t0}}\times 100\%$$

where S_r_ represents the swelling ratio, W_t_ andW_t0_ respectively represent weights of the tablet at a certain time and its original dry weight. The values were averaged over three weight readings for each tablet for minimizing measurement errors.

Since HPMC is a derivative of cellulose obtained by adding hydroxypropyl and methyl groups to substitute primary and secondary hydroxyl groups, three factors, namely, methyl content, hydroxypropyl content, and molecular weight control the final properties and behavior of HPMC [97]. Although average molecular weight determines viscosity in an aqueous media, the physicochemical structures and distribution of substitution groups have a great influence on the final properties of HPMC. Comparing the parameters for HPMC-A and HPMC-C, it can be seen that both HPMC have similar substitution ratio of methyl and hydroxypropyl groups. Viscosity of C is 667 times higher than that of A whereas the molecular weight of C is 8.6 times higher than that of A. This confirms that the molecular weight of HPMC plays a dominant role in changing the viscosity when the polymers have similar chemical structures. When parameters for A and B, which have different percentage of methyl substitution, are compared, viscosity of B is found to be 7–10 times higher than that of A. The methyl substitution ratio for B is approximately half that of A, while the average molecular weight of B is slightly over two times of A. Thus, variations in methyl substitution and molecular weight lead to different viscosity. A possible explanation is that the viscosity of HPMC is influenced by the hydrophobic interactions introduced by methyl groups onto HPMC backbone in different ratios. It seems that hydrophobic associations caused by the methyl groups’ substitution enhance the viscosity of HMPC with the increasing molecular weight.

Due to methyl (hydrophobic) and hydroxypropyl (hydrophilic to some extent) substitution, the network of hydrogen bondings (H-bondings) in cellulose is disrupted. The hydrophobic interactions between methyl groups and water can lead to self-aggregation of HPMC chains. The aggregation is accelerated with aid of interactions with water (H-bonding) at elevated temperature or in a highly plasticized state, which leads to the gelation of HPMC in most cases. HPMC shows more affinity to water molecules, leading to higher water solubility of the polymer. The opposing effects of the hydrophobic methyl groups and hydrophilic hydroxypropyl groups grant HPMC the swellability in the aqueous media. By adjusting the hydrophobic-hydrophilic balance with the suitable ratio of hydroxypropyl and methyl groups, the swellability of HPMC in the aqueous media can be modulated.

The T_g_ of HPMC-A, -B and -C were found to be 178, 188 and 163 °C respectively. These results indicate that the polymer chains are almost immobile at 37 °C, the temperature used in the current experimental studies. Therefore it is impossible to have the formation of systematic aggregation taking place spontaneously except the inherent presence of H-bond network.

When HPMC tablets are soaked in aqueous media, a large amount of water is absorbed into the porous polymeric matrix. The absorbed water acts as a plasticizing reagent and reduces the H-bond inter/intra-chains interactions. As a result, T_g_ of HPMC is reduced drastically. This results in reducing energy barriers that restrict the mobility of the functional groups, side chains and polymer main chains. This allows the polymer chains to change the conformation with greater freedom.

**Figure 23:**
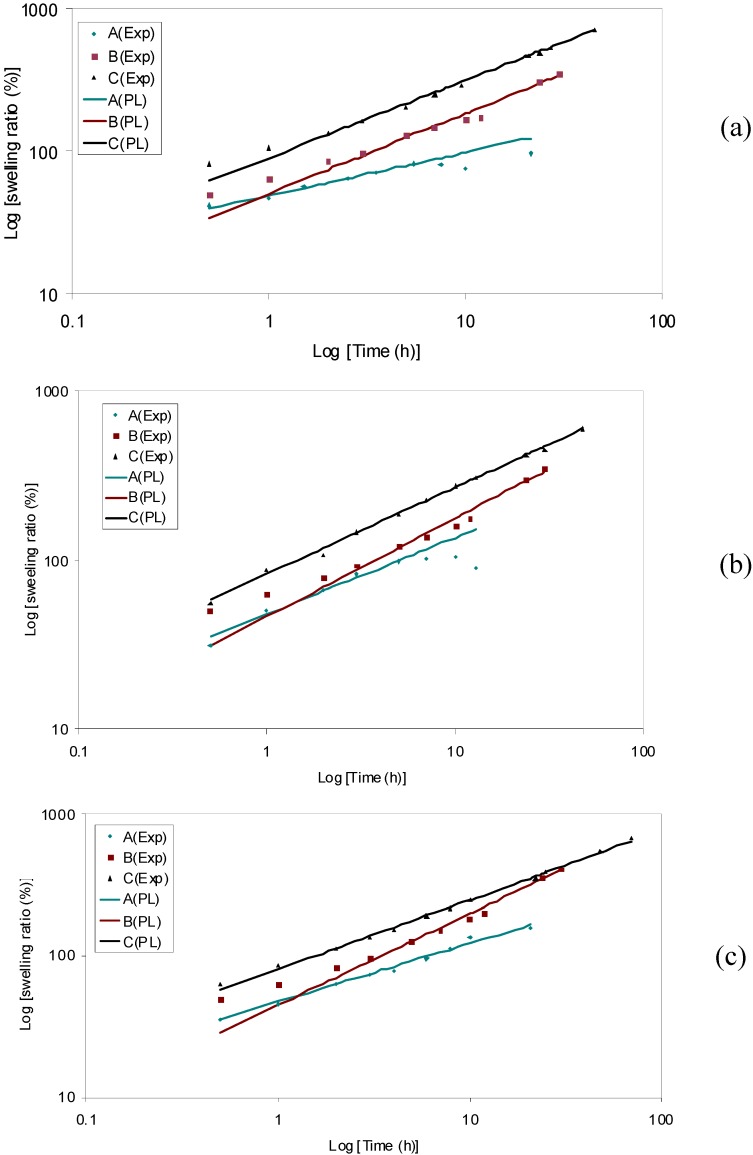
Experimental data (Exp) and power law profiles (PL) defining fluid uptake and swelling ratio for HPMC tablets in: (**a**) water at 37 °C; (**b**) SGF at 37 °C; (**c**) SIF at 37 °C.

As depicted in Figure 23, water, SGF, and SIF have been chosen as media to study the fluid uptake behavior of the different grades of HPMC tablets. Among the three media, HPMC-A tablet could absorb the fluid equal to its original weight. Finally the tablet disintegrated into various portions after 9 h. HPMC-B absorbed a significant amount of water that weighed 3–4 times the weight of the tablet. These tablets kept their shape for 15 h before disintegration. Tablets made of HPMC-C could remain integrated even up to 45 h after absorbing the fluid 6–7 times of their original weight.

It is known that the swelling process is governed by a power law [98] as in Equation (7):

(7) $$S_{r}=a*t^{b}$$

where *Sr* represents the swelling ratio, t means the soaking time and, *a* and *b* are the process related constant parameters. Equation (7) may be expressed as a linear curve by $Log(S_{r})=Log(a)+b*Log(t)$. The experimental data is represented using Equation (7) by obtaining necessary parameters for the best-fit curves. The obtained parameters are listed in Table 9 and the experimental data along with the best-fit lines on log–log scale are shown in Figure 23.

**Table 9:** Power law parameters simulating swelling of HPMC.

Equation	Media	Parameters					
		HPMC A		HPMC B		HPMC C	
		a	b	a	b	A	b
S_r_ = a*T^b^	Water	49.04	0.30	50.21	0.56	89.75	0.54
	SGF	48.19	0.45	46.46	0.58	83.31	0.51
	SIF	46.96	0.41	44.72	0.64	79.15	0.49

HPMC-A, -B and -C show similar trends irrespective of the type of media they are soaked in. The swelling patterns in the three media also show a similar trend with similar swelling rate and final swelling ratio. These results testify that the variants like pH and ionic strength of media have little effect on the swelling behavior in the adopted time scale [99]. The difference in the parameters suggests that water uptake and swelling processes are primarily controlled by the nature of the polymer.

**Figure 24:**
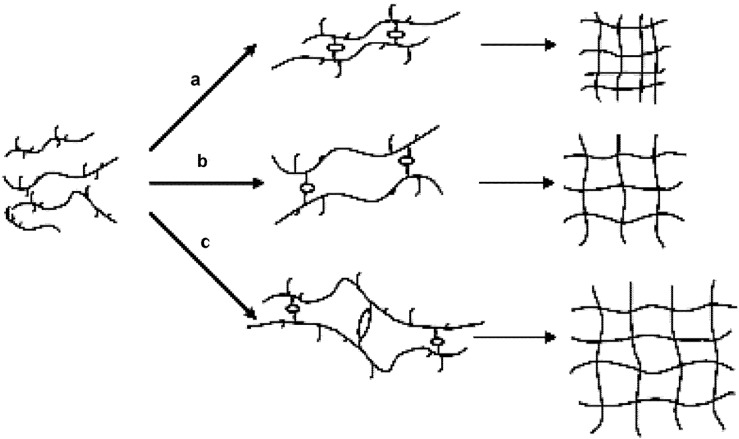
Aggregation patterns for various grades of HPMC having different substitution ratio and molecular weight as: (**a**) network of HPMC-A with small cells; (**b**) network of HPMC-B with larger cells at lower cross-linking density; (**c**) network of HPMC-C with large cells at the highest cross-linking density.

Many factors including particle size and distribution of powder, porosity of the formed tablets, interaction between polymer and solvent, influence the rate at which the fluid media is absorbed into HPMC tablets. However, the determining factors should be the physicochemical properties of HPMC, especially when a large amount of fluid media is absorbed in the swollen state. Hydrophobic interactions between methyl groups and water are the main drivers for the spontaneous swelling process. The self-aggregation behavior of different grades of HPMC is linked to their different chain structures. The ordered network formed is determined by the density of methyl groups in the polymer-fluid system at the swollen state. The possible mechanism involved in the fluid uptake and swelling processes is shown in Figure 24. HPMC-A has the lowest average molecular weight and relatively higher methyl substitution ratio among all the three grades of HPMC. Therefore the physical cross-linking sites formed by the clustering of methyl groups in the swollen state are limited, and the formed network is composed of multiple shorter polymer chains in the medium aggregation scale. The density of physical cross-linking sites is limited by the shorter length of the polymer chains and the fewer total number of methyl groups. Thus, it is believed that the cells in the gelation network are of small sizes. HPMC-B has higher molecular weight range (longer polymer chains) but the lowest substitution ratio of methyl groups. The formed network for this type of HPMC seems to be composed of longer polymer chains with fewer cross-linking sites, resulting in a loosely formed network. HPMC-C has the longest polymer chains, and the highest ratio of methyl group substitution. This induces the formed network with the highest density of physical cross-linking sites.

The size of the formed network cells and the density of the physical cross-linking sites not only determine the strength of the gel layers but also control the disintegration behavior of the direct-compressed tablets with time in the fluid media. Obviously, HPMC-A had the weakest network that disintegrated much earlier than HPMC-B. HPMC-C exhibited the strongest network and stable shape even after absorbing considerable amount of the fluid media.

### 6.2. Drug Release Profiles for Indomethacin-Loaded Tablets

Indomethacin in powder form was mixed with each of the three types of pre-dried HPMC powders in 1:20 ratio, respectively. Each mixture was then compressed at 400 kg/cm^2^ pressure for 1 min at room temperature in tablet maker. The ready tablets were soaked in a 50 mL SIF solution maintained at 37 °C adopting the same procedure as described in the earlier section. Starting from the first half an hour until the 60th h, a 0.25 mL of drug-mixed solution was taken out every 30 min and diluted to 3 mL aqueous for UV measurements to determine the concentration of Indomethacin in the fluid media derived from the immersed tablets. A series of aqueous solution samples with different concentrations of Indomethacin were prepared separately and tested for UV absorption at 265 nm wavelength, and the relationship between the UV readings versus the drug concentrations was set as a standard curve. By comparing the results of UV tests on the collected samples with the standard curve, the release profiles for Indomethacin loaded HPMC tablets were obtained.

**Figure 25:**
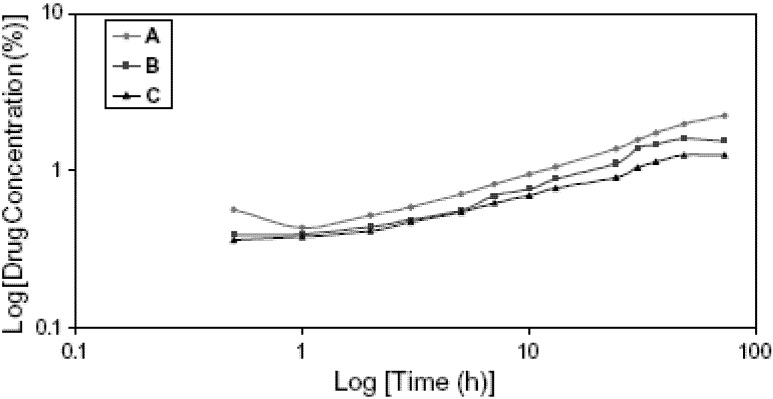
Indomethacin release profiles of HMPC tablets in SIF at 37 °C.

From the release profile in SIF shown in Figure 25, it can be seen that the accumulated amount of Indomethacin in the media increased fast at the initial stages (within the first 10 h) for all the three grades of the HPMC tablets. The drug release rate became approximately constant after 30 h for the tablets made of HPMC-B and HPMC-C, and the same trend was maintained for the remaining time scale. Due to direct-compression technique used for forming the tablets, any variations in the release patterns for the three grades of the HPMC tablets are believed to be linked directly to the changes in the fluid uptake and the swelling process. It may be observed that in Figure 25, the sequence of drug release rate follows the order of HPMC-A > HPMC-B > HPMC-C, which is the reverse of the trend for the swelling and disintegration of HPMC tablets in SIF discussed in the earlier sections.

It is known that the gel barrier is the determining factor of translocation of drug transportation from HPMC matrix [100]. The faster the formation of gelation barrier, the thicker the gel layer is, leading to slower drug release in the early stage. In the latter stage, the thickness of the gel layer and the integration of the HPMC network in the swollen tablets are the key factors in determining the drug release profile in the related time scale.

**Figure 26:**
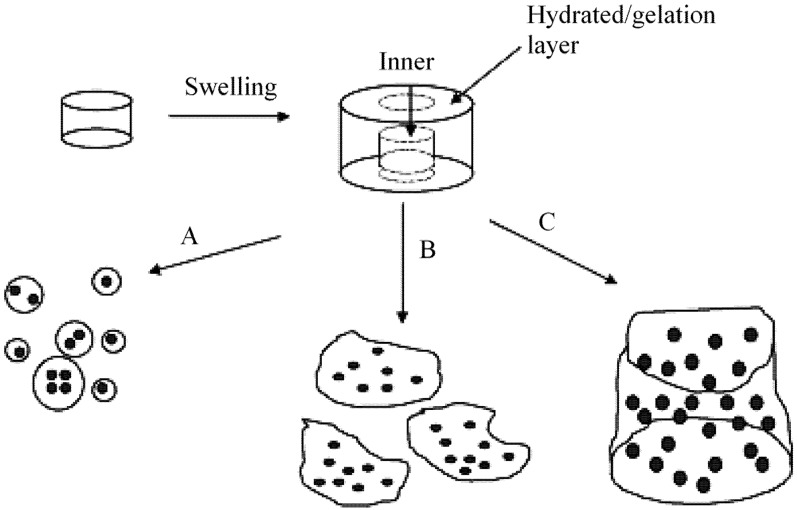
Water uptake process and subsequent disintegration of tablets in the drug release process (A stands for 6 mPa.s HPMC; B for 40–60 mPa.s HPMC and C for 4000 mPa.s HPMC).

Based on the drug release profile, a detail description of the possible mechanism of the process can be depicted as shown in Figure 26.

In the early stage, the drug-loaded tablets absorb a large amount of SIF into the porous matrix. This causes the outer layer to hydrate and convert the part into gel with the assistance of water molecules as plasticizing agents. As time progresses, the outermost layer dissolves either partially or fully into the media, allowing more water molecules to diffuse into the inner core region, which is intact so far, by forming a new gel front. The drug that remains rapped within the disintegrated part is released faster than that from those separated portions of the HPMC gel. The thickness of the gelation layer is rather thin at the early swelling stage, which is also the reason for the faster drug release. As more and more aqueous media is absorbed into the tablets, the gelation layer gets thicker and thicker until it reaches the fully swollen stage, which is the fully saturated and expanded state of the gel. With more water coming in, the fully swollen tablets show different routes in the later stage of drug releasing process. In the case of HPMC-A, the tablets disintegrate into various microgel particles containing drug in small amount at a rather early stage (10 h or so). Due to the smaller sizes of the microgel particles that float in the media in large numbers, the loaded drug is able to easily pass through the thin gel layer and dissolve into the media around. This has resulted in the fastest release rate among the three grades of HPMC. As far as HPMC-B is concerned, after achieving the fully swollen state, the tablets disintegrated into much larger pieces compared to HPMC-A. The much thicker gel layer and smaller specific surface area led to a slower drug release rate after 25 h. During the whole time scale, HPMC-C tablets kept its content integrated; the tablets became fully gelated with a small residual core. The thickest gel barrier hindered the diffusion of the drug trapped within the matrix into the media freely, resulting in the slowest drug release rate.

## 7. Conclusions

The investigations revealed that most salts (except NaI and NaSCN) added to aqueous solutions of HPMC lead to salting-out effects and therefore promote thermogelation of HPMC. NaI and NaSCN, however, showed salting-in effects and suppressed the thermogelation. The effectiveness of anionic species in reducing T_m_ was found to in the same sequence of the Hofmeister series and attributed to their ability to influence water structure. Microcalorimetry results disclosed that the effect of monovalent salts on sol-gel transition was more cooperative than that of multivalent salts. All in all, the gel strength increased in the presence of salting-out salts, while it decreased slightly in the presence of salting-in salts.

Similar to the salts, thermal behavior of HPMC hydrogels was influenced by surfactants. With the addition of SDS lower than 6mM, SDS existed as dissociative ions within the solution and the gelation of HPMC/SDS mixtures occurred at lower temperatures as compared to pure HPMC hydrogels. The addition of SDS higher than 6mM concentrations significantly affected the HPMC gelation; not only the peak shape of the DSC thermograms changed from a single mode to a bimodal but also each thermogram covered a wider range of temperature with reduced height of the first peak. The addition of SDeS in different concentrations had a salt-in effect on HPMC gelation without having any effect on the shape of corresponding thermograms although the thermograms became narrower with increasing the concentration of SDeS. SHS did not significantly affect the sol-gel transition of HPMC hydrogels except for apparently increasing the heat capacity. The anionic (SDS, SDeS, SHS) surfactants increased the energy barrier of the sol-gel transition due to their priority binding to the hydrophobic parts of HPMC inducing polar outshells, which hinder the free access to HPMC chains at elevated temperature. The non-ionic (Triton X-100) surfactant showed much less influence on the gelation of HPMC solution. Difference in the chemical structure and electrostatic interaction between the surfactant and HPMC molecules determined the thermal energy requirements for sol-gel transitions in the ternary mixtures of surfactant/HPMC/water.

The thermoreversible sol-gel transition of HPMC in simulated intestinal/gastric fluids provided an endothermic peak upon heating and an exothermic peak upon cooling using a micro-DSC. Enthalpy and entropy changes were linearly proportional to the HPMC concentration. Similar phenomena were observed for samples in SGF and SIF. Both SGF and SIF do not change the sol-gel transition mechanisms of HPMC, but the temperature at which these sol-gel transitions took place is decreased with the increasing SGF/SIF content. At the same time, enthalpy and entropy changes involved in the sol-gel transition increased. This salting-out effect was the result of the competition for free water molecules between the salt ions and the HPMC chains. Rheological measurements showed that the gel strength of semi-dilute HPMC solutions increased abruptly during the sol-gel transition and eventually reached a plateau upon heating. However, the gel strength decreased gradually upon cooling. Fluorescence experiments confirmed that the sol-gel transition of HPMC solution was driven by hydrophobic association.

The swelling behavior of tablets of all three grades of HPMC in water, SGF, and SIF, all showed very similar trends. It was observed that the percentage substitution of methyl side groups and molecular weight of the polymer determine and control the various patterns of the fluid uptake and the swelling process for the tablets. The drug release profiles for the Indomethacin-loaded tablets showed a reverse trend as compared to the fluid uptake process. However, similar to the fluid uptake process, drug release was also controlled by the size and density of the gel network.
